# Sleep-dependent alterations in anterior cingulate cortex excitation–inhibition balance link alterations in BOLD-CSF coupling to cognitive performance in obstructive sleep apnea

**DOI:** 10.3389/fnagi.2026.1932223

**Published:** 2026-09-11

**Authors:** Youquan Ning, Yilan Li, Zhenliang Xiong, Zehu Sheng, Lian He, Shaoxin Xiang, Yida Hu, Juan Peng, Fajin Lv

**Affiliations:** 1 Department of Radiology, The First Affiliated Hospital of Chongqing Medical University, Chongqing, China; 2 College of Computer Science and Technology, Guizhou University, Guiyang, Guizhou, China; 3 Department of Geriatrics, Laboratory of Research and Translation for Geriatric Diseases, The First Affiliated Hospital of Chongqing Medical University, Chongqing, China; 4 Department of Neurology, The First Affiliated Hospital of Chongqing Medical University, Chongqing, China; 5 MR Scientific Collaboration, United Imaging Healthcare, Shanghai, China

**Keywords:** BOLD-CSF coupling, cognition, excitation/inhibition balance, GABA, Glx, obstructive sleep apnea

## Abstract

**Objective:**

The roles of cortical glymphatic dysfunction and excitation-inhibition imbalance in cognitive impairment associated with obstructive sleep apnea (OSA) require further study. This study aimed to investigate sleep-related glymphatic function and neurotransmitter changes in OSA and their relationships with cognition.

**Methods:**

Eighty seven participants (50 OSA, 37 controls) completed a pre-sleep MRI, followed by an overnight PSG recording, and a post-sleep MRI to assess sleep-dependent glymphatic and neurotransmitter changes. Resting-state blood-oxygen-level-dependent (BOLD) signals and cerebrospinal fluid (CSF) signals (BOLD-CSF coupling), an indirect glymphatic-related imaging proxy, were assessed in the global cortex, anterior cingulate cortex (ACC), posterior cingulate cortex (PCC), and default mode network (DMN) cortex. MRS-derived gamma-aminobutyric acid (GABA) and glutamate/glutamine (Glx) levels were measured in the ACC and PCC as indirect neurochemical measures of excitation-inhibition (E/I) balance. General linear models, linear mixed-effects models, correlation analyses, and moderation analyses were employed to examine group differences, overnight changes in BOLD-CSF coupling and neurotransmitters, and their relationships with cognitive performance.

**Results:**

In the moderate-to-severe OSA group, pre-sleep global and ACC-BOLD-CSF coupling were weaker than in controls, and ACC-GABA showed a group × time interaction (η_p_^2^ = 0.105, *p*-FDR = 0.049), with a significant overnight decrease observed only in controls (Cohen’s d = 0.75, *p*-FDR = 0.007). Greater magnitudes of overnight change in both ACC-BOLD-CSF coupling and ACC-E/I were independently associated with worse cognition. ACC-E/I change also moderated the coupling-cognition relationship (*β* = 0.590, *p* = 0.015), with a significant negative association only at smaller magnitudes of E/I change (*β* = −0.729, *p* = 0.002).

**Conclusion:**

Our findings suggest that OSA severity is associated with ACC-specific BOLD-CSF decoupling and a disrupted overnight decline in ACC-GABA. The magnitude of overnight E/I variation moderated the association between BOLD-CSF coupling variation and cognitive performance. These preliminary findings highlight the potential relevance of ACC-BOLD-CSF coupling and neurochemical alterations to OSA-related cognitive impairment. Future studies with larger sample sizes are needed to confirm these findings.

## 1 Introduction

Obstructive sleep apnea (OSA), characterized by recurrent arousals and chronic intermittent nocturnal hypoxia, is a prevalent yet under-diagnosed sleep-related breathing disorder (Chan et al., 2019; Shieu et al., 2022). It is associated with excessive daytime sleepiness and impaired quality of life (Pascoe et al., 2022). More importantly, OSA may represent an early but potentially modifiable risk factor for cognitive decline (Marchi et al., 2023; Pase et al., 2023; Ungvari et al., 2025) and neurodegenerative diseases such as Alzheimer’s disease (Liguori et al., 2017), Parkinson disease (Nepozitek et al., 2025), and cerebral small-vessel disease (Chokesuwattanaskul et al., 2020). However, the neuropathological mechanisms linking OSA to cognitive impairment remain unknown.

The glymphatic system is a highly organized perivascular transport network that facilitates convective exchange between cerebrospinal fluid (CSF) and interstitial fluid and mediates the clearance of metabolic waste and interstitial solutes through astrocytic aquaporin-4 (AQP4) (Iliff et al., 2012). Glymphatic failure has been proposed as a final common pathway leading to dementia (Nedergaard and Goldman, 2020). It is well established that glymphatic function exhibits clear circadian fluctuations, and fragmentation of sleep architecture in OSA patients can impair this process, which is most efficacious during slow-wave sleep (Hablitz et al., 2020; Vasciaveo et al., 2023; Lin et al., 2024). Previous quantitative MRI-based studies have shown that patients with OSA have subcortical glymphatic dysfunction, characterized by a reduced diffusion tensor image analysis along the perivascular space (DTI-ALPS) index, increased perivascular space (PVS) burden, and elevated free water in white matter (FW-WM) compared with healthy controls (HCs). Effective interventions such as continuous positive airway pressure (CPAP) and uvulopalatopharyngoplasty (UPPP) can improve glymphatic function and cognitive performance (Lin et al., 2024; Sommer et al., 2025). Although DTI-ALPS is widely used to assess glymphatic function indirectly, recent evidence suggests that it has limited correspondence with contrast-enhanced MRI measures of cortical tracer transport and may be confounded by age- and pathology-related white matter microstructure (Mossige et al., 2026; Li Y. et al., 2025). Given the importance of cortical networks in cognitive function, alterations in cortical glymphatic function in OSA remain poorly understood and warrant further investigation. Recently, Li et al. reported predominantly anterior cortical glymphatic dysfunction in OSA using resting-state blood oxygen level-dependent–cerebrospinal fluid (BOLD-CSF) coupling. This fMRI-based measure has been proposed as a noninvasive imaging marker of cortical glymphatic-related function (Fultz et al., 2019). The cortical alterations were associated with disease severity and cognitive impairment, and were reversible following UPPP (Li Y. et al., 2025). However, previous studies have not adequately characterized dynamic changes across the sleep–wake cycle, particularly at the global or macro-regional cortical levels (Jiang et al., 2023; Li Y. et al., 2025). Higher-order brain networks, particularly the default mode network (DMN), also remain insufficiently explored. The DMN, a core substrate for higher-order cognition, shows early vulnerability to Aβ aggregation years before the clinical diagnosis of neurodegenerative disease. This preferential Aβ deposition has been associated with reduced global brain activity in the same regions and may be related to impaired glymphatic function (Han et al., 2023; Cui et al., 2025). Therefore, studying cortical glymphatic function in the DMN and its subregions may provide a basis for understanding OSA-related cognitive impairment.

Notably, glymphatic function has been associated with neurotransmitter homeostasis. AQP4-mediated glymphatic clearance is influenced by gamma-aminobutyric acid (GABA) signaling (Wu et al., 2020, 2023). Evidence from *in vivo* magnetic resonance spectroscopy (MRS) studies suggests that dysregulation of inhibitory and excitatory neurotransmitter systems involving GABA and glutamate (Glu) may be involved in OSA pathophysiology. GABA/Glu-targeted therapies may also influence the course of OSA (Kaczmarski et al., 2023). Previous MRS-based studies have reported reduced GABA and increased Glu in the insular cortex (Macey et al., 2016) and thalamus/putamen (Sarma et al., 2016), as well as GABA deficiency in the dorsolateral prefrontal cortex (PFC) (Pereira et al., 2017). Nevertheless, the aforementioned studies did not further evaluate their specific associations with cognitive performance. Excitation-inhibition (E/I) imbalance in the ACC and PCC, indirectly assessed using MRS-derived GABA and Glu measures, has been associated with cognitive impairment (Fu et al., 2023; Tseng et al., 2024; Velu et al., 2024). However, research into the ACC and PCC using MRS, and into the relationship between the glymphatic system and neurotransmitters in the cortex, remains poorly understood in OSA.

Building upon this background, the present study assessed BOLD-CSF coupling (global, DMN, ACC, and PCC) and MRS-derived neurotransmitter concentrations (ACC and PCC) using pre-sleep MRI. Overnight PSG was then performed, and the same imaging measurements were repeated during post-sleep MRI the following morning. This protocol enabled the assessment of overnight changes and their associations with OSA severity and cognitive performance. We hypothesized that (a) OSA severity would be associated with region-specific alterations in BOLD-CSF coupling and neurochemical measures; (b) these alterations would be associated with cognitive performance; and (c) within the ACC, BOLD-CSF coupling and neurochemical measures would show an interaction in relation to cognitive performance.

## 2 Materials and methods

### 2.1 Ethics declarations and study participants

The study was conducted in accordance with the Declaration of Helsinki and approved by the Institutional Ethics Committee (IRB No. 2024-145-01). All participants provided written informed consent.

**Figure 1:**
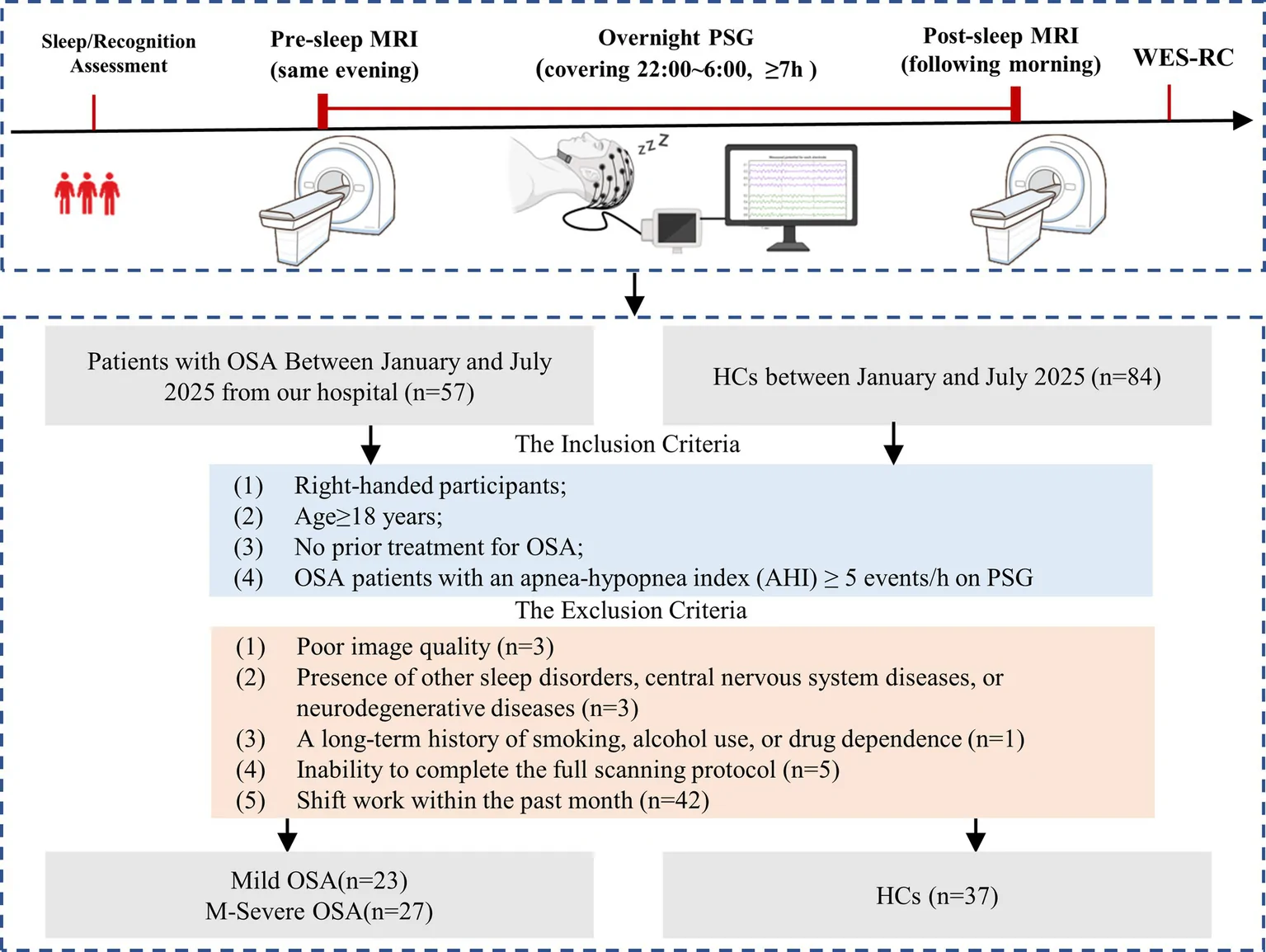
Flowchart of the study workflow and patient recruitment. PSG, polysomnography; WMS-RC, Wechsler Memory Scale–Revised, Chinese version; OSA, obstructive sleep apnea; HCs, healthy controls.

Each participant underwent pre-sleep MRI on the same evening before PSG. Overnight PSG was then conducted for at least 7 h, covering the period from 22:00 to 06:00. Post-sleep MRI was subsequently performed from 06:30 to 07:30 the following morning. Participants were required to refrain from sleep-altering substances and caffeinated beverages for 24 h before PSG and MRI. They were instructed to remain awake throughout the scanning session. After each scan, participants were asked whether they had remained awake or fallen asleep during the preceding scan. The study workflow and participant recruitment are summarized in Figure 1. Multimodal imaging included resting-state functional MRI (rs-fMRI) and Mescher-Garwood point-resolved spectroscopy (MEGA-PRESS) sequence. Rs-fMRI was used to derive BOLD-CSF coupling as an indirect glymphatic-related imaging proxy. MEGA-PRESS was used to quantify inhibitory and excitatory neurotransmitter levels indirectly and to compute the excitatory/inhibitory (E/I) ratio as a proxy for E/I balance in the ACC and PCC. Inclusion criteria were as follows: (1) right-handedness; (2) age ≥ 18 years; (3) no prior treatment for OSA; and (4) an apnea-hypopnea index (AHI) ≥ 5 events/h on PSG for patients with OSA. Exclusion criteria were as follows: (1) poor image quality (*n* = 3); (2) presence of other sleep disorders, central nervous system diseases, or neurodegenerative diseases (*n* = 3); (3) a long-term history of smoking, alcohol use, or drug dependence (*n* = 1); (4) inability to complete the full scanning protocol (*n* = 5); and (5) shift work within the past month (*n* = 42).

### 2.2 Clinical and neuropsychological assessments

Global cognitive performance was evaluated using the standardized Montreal Cognitive Assessment (MoCA), a 30-point test covering multiple cognitive domains. Administration and scoring followed standard protocols.

Domain-specific scores were derived for memory, visuospatial ability, language, attention, and executive function, as previously described (Vogel et al., 2015; Liew, 2025). The component items for each cognitive domain are detailed in Supplementary eTable 1. Higher scores indicate better cognitive performance, and scores < 26 were considered indicative of possible cognitive impairment. To further characterize memory-related function, the Wechsler Memory Scale-Revised (WMS-RC; Chinese version) was administered (Elwood, 1991). The age-adjusted total score of the WMS-RC was defined as the Memory Quotient (MQ), with higher scores indicating superior memory function. The WMS-RC comprises a battery of subtests designed to assess three memory domains: long-term memory (counting 1–100, counting 100–1, and accumulation subtests), short-term memory (recognition, pictures, regenerate, association, touch, and understand subtests), and transient memory (digit span) (Wan et al., 2022). Sleep questionnaires included (1) the Pittsburgh Sleep Quality Index (PSQI) for overall sleep quality; (2) the Morningness-Eveningness Questionnaire (MEQ) for circadian preferences; and (3) the STOP-BANG questionnaire for obstructive sleep apnea risk. Emotional states were evaluated using the Self-Rating Depression Scale (SDS) and the Self-Rating Anxiety Scale (SAS).

### 2.3 Polysomnography

Participants underwent standardized in-hospital PSG using the 80-channel Grael PSG system (Compumedics, Abbotsford, VIC, Australia). The recordings included electroencephalogram, electrooculogram, electromyogram, electrocardiogram, airflow, respiratory effort, oxygen saturation, and limb movements. Sleep stages were scored following the American Academy of Sleep Medicine guidelines. Key sleep parameters included sleep architecture, AHI, mean and lowest O₂ saturation, arousal index, and periodic limb movement index.

### 2.4 fMRI acquisition and preprocessing

#### 2.4.1 fMRI acquisition

All MRI data were acquired using the same 3 T MRI scanner (uMR790, United Imaging Healthcare, Shanghai, China) equipped with a 32-channel head coil. High-resolution 3D T1-weighted structural MRI was acquired with the following parameters: TR/TE = 7/2.3 ms; field of view (FOV) = 256 × 256 mm^2^; matrix = 265 × 256; slice thickness = 1.0 mm; number of slices = 180; flip angle = 8°; and voxel size = 1 × 1 × 1 mm^3^. Resting-state fMRI was acquired using a gradient-echo planar imaging sequence with the following parameters: TR/TE = 2000/30 ms; flip angle = 70°; thickness/gap = 3.7/0 mm; acquisition matrix = 64 × 64; FOV = 237 × 237 mm^2^; number of axial slices = 42; and number of time points = 200 (7.09 min).

#### 2.4.2 fMRI data preprocessing for BOLD and CSF signal extraction

Resting-state fMRI data were preprocessed using SPM12 and DPABI version 6.1,^1^ implemented in MATLAB R2019b (MathWorks, Natick, MA, USA), following previous studies. The preprocessing steps included: (1) removal of the first 10 volumes for signal equilibration; (2) slice-timing correction and realignment for head-motion correction; (3) exclusion of participants with head translation >2.5 mm and/or rotation >2.5°; and (4) removal of linear temporal trends and band-pass filtering (0–0.01 Hz). CSF signal preprocessing followed the above procedures, but without motion correction. We skipped nuisance regression analysis of the BOLD signal, CSF signal, and motion parameters to maintain consistency with previous BOLD-CSF coupling studies and to avoid attenuation of the global BOLD signal or loss of critical hemodynamic information (Fultz et al., 2019; Li Y. et al., 2025; Han et al., 2021). For the BOLD signal, spatial Gaussian kernel smoothing (full-width at maximum, 4 mm) was applied to improve the signal-to-noise ratio.

The global BOLD signal was extracted from the preprocessed rs-fMRI data using the binary mask of cortical gray matter defined by the Automated Anatomical Labeling 2 (AAL2) atlas (Rolls et al., 2015). The DMN signal was extracted from the 7-network functional parcellation proposed by Yeo et al. and was further restricted to the cortical mask (Yeo et al., 2011). To avoid spatial blurring during registration, all fMRI signal analyses were performed within individual gray matter masks without transformation to standard space. The DMN is functionally heterogeneous, and previous rs-fMRI studies have reported reduced functional connectivity between anterior DMN regions and posterior hubs, such as the PCC, in OSA (Chen et al., 2016, 2018; Li et al., 2016). We therefore investigated the anterior and posterior DMN subdivisions and their BOLD-CSF coupling patterns in OSA. Anatomical regions of interest (ROIs), including the ACC, PCC, and precuneus, were defined according to the AAL2 atlas. To enhance network specificity, these anatomical ROIs were constrained by the cortical DMN mask. The resulting DMN-constrained anterior and posterior ROIs were referred to as the ACC-BOLD ROI and PCC-BOLD ROI, respectively (Supplementary eFigure 1A). Regional BOLD signals were then extracted from these ROIs for BOLD-CSF coupling analyses. For CSF signal extraction, experienced radiologists manually segmented the CSF mask on the bottom slice of the rs-fMRI acquisition and confirmed its location using high-resolution T1-weighted images. The accuracy of ROI segmentation was cross-checked by a senior radiologist. The mean CSF time series was then extracted for each participant (Supplementary eFigure 1B).

We calculated the cross-correlation function between BOLD and CSF signals across time lags of −10 s to 10 s (Zhang et al., 2024; Li Y. et al., 2025). For each subject, the coupling strength was determined by the negative peak at a + 4 s lag, corresponding to the strongest functional correlation. Statistical significance was assessed using a permutation test. BOLD and CSF signals from different participants were randomly paired, and the cross-correlation was recalculated 10,000 times to generate a null distribution. Furthermore, we calculated the cross-correlation between the CSF signal and the negative first-order derivative of the BOLD signal (Supplementary eFigure 1C). To examine regional specificity, we calculated cross-correlations between the CSF signal and BOLD signals from the DMN, ACC, and PCC. The resulting coefficients were termed DMN-BOLD-CSF, ACC-BOLD-CSF, and PCC-BOLD-CSF coupling, respectively (Supplementary eFigure 1D).

### 2.5 MRS data acquisition and preprocessing

#### 2.5.1 MRS data acquisition

**Figure 2:**
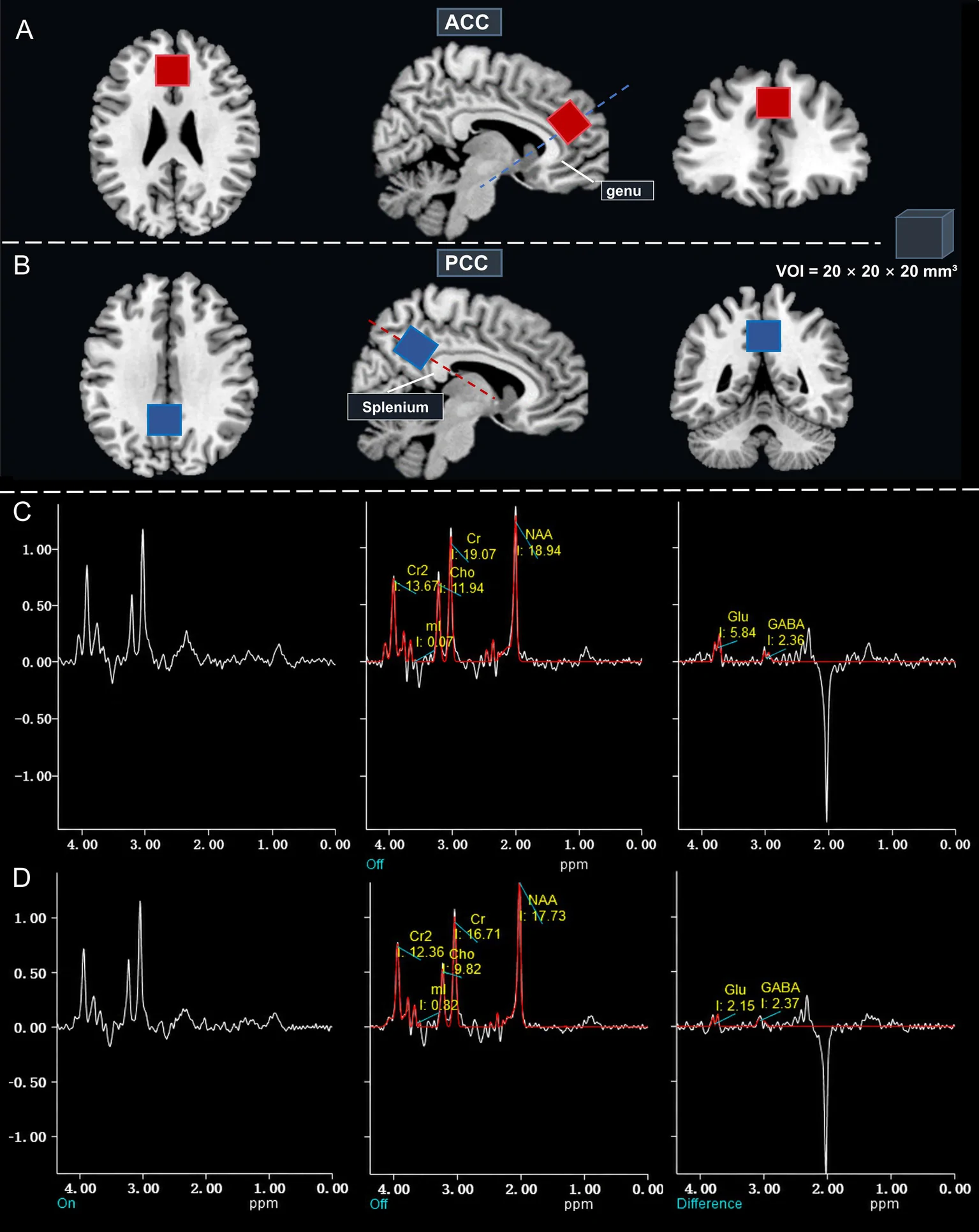
MRS voxel placement and representative MEGA-PRESS spectra. **(A,B)** Representative axial, sagittal, and coronal T1-weighted images showing placement of the 20 × 20 × 20 mm^3^ VOIs in the ACC (red) and PCC (blue). The ACC VOI was positioned superior to the genu, whereas the PCC VOI was aligned with the posterior border of the splenium. Dashed lines were used to assist with voxel localization and indicate planes perpendicular to the VOI. **(C,D)** Representative ACC **(C)** and PCC **(D)** MEGA-PRESS spectra generated by the vendor-provided spectroscopy analysis software, including the edit-ON spectrum (left), the edit-OFF spectrum with Gaussian peak fitting (middle), and the ON-minus-OFF edited difference spectrum (right). The edited signals at approximately 3.0 and 3.75 ppm were labeled by the software as GABA and “Glu”, respectively. Because glutamate and glutamine could not be reliably separated, the latter signal is reported as Glx (glutamate/glutamine) throughout the manuscript. *Cr*, creatine; *GABA*, gamma-aminobutyric acid; *NAA*, N-acetylaspartate; *VOI*, volume of interest.

Single-voxel proton MRS was performed on the same 3 T MR scanner. Foam padding was utilized to minimize head motion. The ACC and PCC voxels (20 × 20 × 20 mm^3^) were centered on the midline sagittal T1-weighted images and confirmed across axial and coronal views. Post-sleep voxel placement was performed by the same operator using the pre-sleep axial, sagittal, and coronal volume of interest (VOI) views as references. The ACC voxel was placed superior to the genu of the corpus callosum, with its posterior edge 2 mm from the callosal margin (Egerton et al., 2023). The PCC voxel was aligned sagittally with the posterior border of the splenium, maintaining a 2-mm margin from the splenium and occipitoparietal fissure to minimize chemical-shift displacement (Bednarik et al., 2021) (Figures 2A,B). Detailed specification of VOI placement facilitated reproducible positioning by the same operator (Park et al., 2016). GABA and glutamate/glutamine (Glx) were detected using the MEGA-PRESS sequence with the following parameters: TR/TE = 2,000/69 ms; spectral width = 2,000 Hz; and number of data points = 1,024. Interleaved editing pulses were applied at 1.9 ppm (editing-on) and 7.5 ppm (editing-off) with 100 on-and-off averages, yielding 200 total transients per VOI. Each voxel acquisition lasted 15 min 40 s, with a total acquisition time of approximately 31 min. Water suppression was achieved using the Water Suppression Enhanced Through T1 Effect (WET) module. Unsuppressed water reference spectra were also acquired at each VOI for eddy-current correction. The shimming outcome was considered acceptable when the creatine (Cr) peak FWHM was below 7 Hz. MRS acquisition and analysis in the present study adhered to the minimum reporting standards for MRS (Lin et al., 2021). The completed MRS reporting checklist is provided in Supplementary eTable 2.

#### 2.5.2 MRS data processing and quality control

Spectral postprocessing and quantification were performed on a uWS-MR workstation (version 2.1; United Imaging Healthcare) using the vendor-provided Spectroscopy Analysis Tool with Gaussian line-shape fitting. The vendor-supplied basis set included the following metabolites: NAA, creatine, phosphocreatine (Cr2), choline, myoinositol, GABA, and Glu. The main processing pipeline was as follows. First, an SNR-based coil combination was used to merge signals from the 32-channel head coil. Automatic frequency alignment and zero- and first-order phase correction were then applied to each transient to minimize frequency and phase inconsistencies. The corrected free induction decays (FIDs) were averaged to improve the SNR. The averaged time-domain signal was zero-filled from 1,024 to 2048 data points to increase digital resolution. Residual water was suppressed using low-pass filtering. Finally, a Fourier transform was performed to obtain the frequency-domain spectra. The interleaved edit-on and edit-off spectra were reconstructed and averaged separately. The averaged edit-off spectrum was then subtracted from the corresponding edit-on spectrum to generate the difference spectrum. GABA (~3.0 ppm) and Glu (~3.75 ppm) signals were quantified from the edited difference spectrum using Gaussian line-shape fitting (Figures 2C,D). Although the software labeled the signal as “Glu,” evidence suggests that it includes overlapping contributions from glutamine and is therefore more appropriately interpreted as Glx (glutamate/glutamine) (Ramadan et al., 2013; Zhang and Shen, 2016; Bell et al., 2021). Accordingly, the E/I ratio was defined as Glx/GABA in the present study. The fractions of gray matter, white matter, and CSF within each MRS voxel were obtained using SPM12 on T1-weighted images. To quantify the spatial reproducibility of voxel placement across sessions, the post-sleep T1-weighted images were linearly co-registered to the pre-sleep T1 images in SPM12. The respective MRS voxel masks were then transformed into the common anatomical space. The spatial overlap between the pre- and post-sleep voxel locations was evaluated using the Dice similarity coefficient (Appendix 4). A Dice value > 0.80 was considered indicative of good spatial reproducibility.

Data quality control adhered to the minimum reporting standards for MRS (Lin et al., 2021). Spectral reliability was assessed using visual inspection, signal-to-noise ratio (SNR), and full width at half maximum (FWHM). Data exclusion criteria were as follows: (1) visual inspection: each fitted spectrum was visually inspected during the scan. Spectra with gross artifacts, distorted peak shapes, abnormal baselines, or unsuccessful peak identification were excluded, and rescanning was performed when necessary; (2) NAA SNR < 10 (Lewis et al., 2020); and (3) NAA FWHM ≥ 7 Hz.

### 2.6 Statistical analysis

Normality was assessed using the Kolmogorov–Smirnov test. Due to non-normal distributions in several demographic and clinical variables, group comparisons of continuous variables were performed using the Kruskal-Wallis test, followed by Dunn’s *post hoc* test with false discovery rate (FDR) correction. Categorical variables were compared using chi-square tests. Data are presented as mean ± SD for continuous variables and as frequencies (percentages) for categorical variables.

General linear models with age, gender, education, and BMI as covariates were used to examine group differences in pre- and post-sleep BOLD-CSF coupling and neurotransmitter levels. FDR correction was applied across the four brain regions. Partial correlation analyses were performed with adjustment for age, gender, education, and BMI. These analyses examined (1) associations of sleep parameters with pre-sleep BOLD-CSF coupling and neurochemical measures; (2) relationships between excitatory and inhibitory neurotransmitter measures within the ACC and PCC; and (3) associations between pre-sleep BOLD-CSF coupling and neurotransmitter concentrations. FDR correction was applied for multiple comparisons.

Linear mixed-effects models (“nlme” package, version 3.1.168) were used to examine the effects of group (HCs, mild OSA, moderate-to-severe OSA), time (pre-sleep, post-sleep), and their interaction on imaging indicators, with subjects as random intercepts and age, gender, education, and BMI as covariates. FDR correction was applied separately across four BOLD-CSF coupling regions and four neurotransmitter measures (ACC/PCC GABA and Glx). Significant interactions (*p*-FDR < 0.05) were followed by simple main-effects analyses, with the effect of time within each group examined using paired *t*-tests.

Overnight changes in imaging indicators were primarily quantified as absolute-change scores (dif = |post − pre|; difBOLD-CSF coupling and difE/I), which reflect the magnitude of overnight variation. Multiple linear regression analyses examined the associations of these absolute-change scores with MoCA scores. The models were adjusted for age, gender, education, BMI, and the corresponding pre-sleep values (pre-sleep BOLD-CSF coupling and pre-sleep E/I ratio). A moderation analysis was subsequently conducted to test the interaction between difBOLD-CSF coupling and difE/I in relation to MoCA scores. Continuous variables were standardized to z scores before model fitting. Significant interactions were probed using simple slope analysis (±1 SD) and the Johnson–Neyman technique. As a complementary analysis, the regression and moderation analyses were repeated using signed overnight change scores (post-sleep − pre-sleep), which preserve the direction of change. Positive and negative values indicate overnight increases and decreases, respectively.

Analyses were performed in R (version 4.5.3) and SPSS (version 27.0, IBM), with statistical significance defined as *p* < 0.05 (two-tailed). Effect sizes were reported as ε^2^ for Kruskal–Wallis tests, Cohen’s *d* for pairwise comparisons, partial η^2^ (ηp^2^) for GLM/LMM, and ΔR^2^ and standardized β with 95% CIs for moderation analyses.

## 3 Results

### 3.1 Clinical and demographic characteristics

Table 1 summarizes the clinical and demographic information of the patients with OSA and HCs. Among the 87 enrolled participants, 37 were classified into the HCs group (mean age, 34.97 ± 13.10 years; 24 males), 23 into the mild OSA group (mean age, 35.91 ± 12.8 years; 17 males), and 27 into the moderate-to-severe OSA group (mean age, 41.52 ± 9.8 years; 24 males). BMI differed significantly among the groups (BMI: ε^2^ = 0.174 [95% CI: 0.048, 0.364]; *p* < 0.001) and increased progressively with OSA severity. There were no significant differences among the three groups in gender distribution, age, years of education, or lifestyle habits (coffee/tea intake, exercise, smoking, and alcohol consumption) (all *p* > 0.05). Consequently, age, gender, education, and BMI were included as covariates in all subsequent neuroimaging analyses. PSG results showed that, compared with HCs, patients with OSA had significantly lower mean and lowest oxygen saturation and a significantly higher arousal index (ε^2^ range: 0.118–0.642; all *p* < 0.001). Regarding sleep macrostructure, wake time (*p* = 0.010) and N1 sleep duration (*p* < 0.001) increased, while N3 sleep duration (*p* = 0.001) decreased as OSA severity progressed. Total sleep time and sleep efficiency remained comparable across all groups (all *p* > 0.05).

**Table 1:** Demographic information for all participants at baseline.

Variable	HCs (*N* = 37)	Mild (*N* = 23)	M-Severe (*N* = 27)	*p* value
Age (y)	34.97 ± 13.10	35.91 ± 12.80	41.52 ± 9.80	0.056
Gender, male (*n*, %)	24 (64.9)	17 (73.9)	24 (88.9)	0.092
Education (y)	16.22 ± 2.98	16.22 ± 3.32	14.96 ± 3.58	0.215
Coffee (times/week)	1.70 ± 2.27	2.04 ± 2.60	0.63 ± 1.08	0.336
Tea (times/week)	1.59 ± 2.10	1.87 ± 1.94	2.33 ± 2.66	0.575
Exercise				0.153
Non-exercise	9 (24.3)	9 (39.1)	15 (55.6)	
<2.5 h/week	12 (32.4)	5 (21.7)	5 (18.5)	
≥2.5 h/week	16 (43.2)	9 (39.1)	7 (25.9)	
Alcohol (*n*, %)				0.147
Non-drink	13 (35.1)	10 (43.5)	5 (18.5)	
0–1 times/week	22 (59.5)	9 (39.1)	15 (55.6)	
2–3 times/week	2 (5.4)	2 (8.7)	3 (11.1)	
≥3 times/week	0 (0.00)	2 (8.7)	4 (14.8)	
Ever smoker (*n*, %)	5 (13.5)	7(30.4)	9 (33.3)	0.134
BMI (kg/m^2^)	22.82 ± 2.51	24.88 ± 2.86^b^	26.16 ± 3.81^a^	<0.001
Questionnaire				
PSQI	6.46 ± 3.81	6.73 ± 2.76	7.71 ± 3.29	0.471
STOP-BANG	1.72 ± 1.17	2.23 ± 1.19	4.00 ± 1.29^a^^,^^c^	<0.001
MEQ	51.88 ± 11.14	51.36 ± 12.77	55.08 ± 10.62	0.553
SAS	30.13 ± 5.50	30.50 ± 4.42	32.50 ± 5.70	0.047
SDS	32.14 ± 9.07	30.68 ± 6.38	32.96 ± 6.71	0.178
MoCA	27.22 ± 1.79	26.78 ± 1.59	25.04 ± 2.70^a^	0.002
Memory	10.39 ± 1.25	10.74 ± 1.21	9.78 ± 1.25^c^	0.022
Visuospatial ability	3.53 ± 0.65	3.48 ± 0.67	3.33 ± 0.92	0.841
Language	4.81 ± 0.40	4.70 ± 0.56	4.22 ± 0.85^a^	0.006
Attention	3.72 ± 0.51	3.78 ± 0.52	3.93 ± 0.27	0.130
Executive function	4.56 ± 0.56	4.30 ± 0.63	3.74 ± 0.94^a^^,^^c^	<0.001
WMS-RC test (scores)				
MQ	110.53 ± 14.56	118.82 ± 12.23	114.83 ± 11.48	0.313
Sleep architecture				
AHI	1.67 ± 1.23	8.19 ± 2.37^b^	41.10 ± 17.74^a^^,^^c^	<0.001
TST (min)	366.40 ± 53.83	351.13 ± 106.43	366.65 ± 58.57	0.785
Efficiency (%)	85.82 ± 9.23	78.66 ± 20.98	83.93 ± 12.26	0.693
Wake time (min)	34.26 ± 39.30	74.30 ± 81.06^b^	50.98 ± 37.98	0.010
Mean O_2_ (%)	96.30 ± 1.00	95.91 ± 0.67	93.13 ± 3.38^a^^,^^c^	<0.001
Lowest SpO₂ (%)	89.58 ± 3.47	85.48 ± 4.26^b^	72.67 ± 9.44^a^^,^^c^	<0.001
REM (min)	63.18 ± 23.37	57.76 ± 28.87	50.29 ± 23.17	0.206
N1 (min)	24.26 ± 19.31	37.46 ± 21.95	74.98 ± 52.91^a^	<0.001
N2 (min)	206.78 ± 46.27	192.57 ± 70.26	206.13 ± 51.47	0.930
N3 (min)	68.58 ± 27.58	62.17 ± 31.16^b^	32.90 ± 26.53^a^	0.001
Arousal index (events/h)	8.95 ± 4.44	11.98 ± 5.63	18.37 ± 10.15^a^	<0.001

Continuous variables are presented as mean ± SD and were compared using the Kruskal–Wallis test. Categorical variables are presented as numbers (percentages) and were compared using chi-square tests. HCs, healthy controls; M-Severe, moderate-to-severe; BMI, body mass index (calculated as weight in kilograms divided by height in meters squared); PSQI, Pittsburgh Sleep Quality Index; STOP-BANG, Snoring, Tiredness, Observed apnea, high blood Pressure, BMI, Age, Neck circumference, and Gender; MEQ, Morningness-Eveningness Questionnaire; SAS, self-rating anxiety scale; SDS, self-rating depression scale; MoCA, Montreal Cognitive Assessment; MQ, memory quotient; WMS-RC, Wechsler Memory Scale–Revised, Chinese version; AHI, apnea-hypopnea index; TST, total sleep time; REM, rapid eye movement; N1/N2/N3, non-rapid eye movement sleep stages 1/2/3.

a *p* < 0.05 versus the HCs group after FDR correction.

b *p* < 0.05 versus the HCs group after FDR correction.

c *p* < 0.05 versus the Mild OSA group after FDR correction.

Global cognitive performance differed significantly among groups (MoCA, ε^2^ = 0.123 [95% CI: 0.019, 0.299]; *p* = 0.002). Domain-specific analyses showed significant group differences in memory (ε^2^ = 0.068; *p* = 0.022), language (ε^2^ = 0.099; *p* = 0.006), and executive function (ε^2^ = 0.146; *p* < 0.001). Additional MoCA domain and pairwise group comparisons are summarized in Table 1. Additionally, STOP-BANG scores differed significantly among groups (ε^2^ = 0.444; *p* < 0.001), with values increasing alongside disease severity. No significant inter-group differences were observed for PSQI, SDS, or MQ scores (all *p* > 0.05).

### 3.2 Comparisons of imaging characteristics among groups and relationships with PSG characteristics

At the pre-sleep baseline, after adjustment for age, sex, education, and BMI, significant between-group differences were observed in Global-BOLD-CSF coupling (*F* = 4.946, η_p_^2^ = 0.110 [95% CI: 0.008, 0.240], *p*-FDR = 0.038) and ACC-BOLD-CSF coupling (*F* = 4.038, η_p_^2^ = 0.092 [95% CI: 0.001, 0.217], *p*-FDR = 0.043).

**Figure 3:**
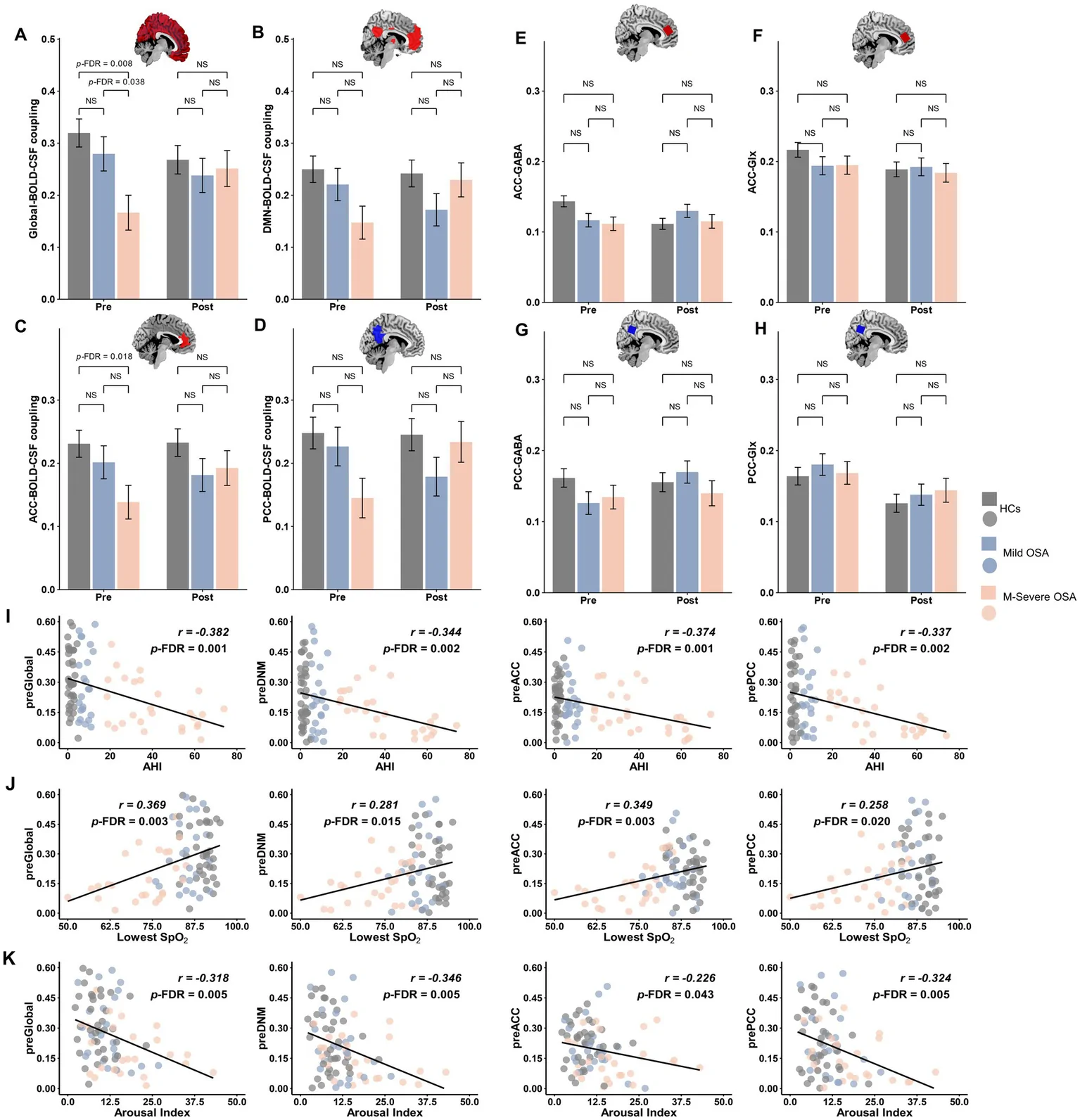
Comparisons of neuroimaging markers among groups and associations with PSG characteristics. **(A–D)** Changes in global and regional BOLD-CSF coupling before and after sleep in patients with OSA of varying severity and healthy controls. **(E–H)** Comparisons of Glx and GABA concentrations before and after sleep in the ACC **(E,F)** and PCC **(G,H)** among patients with OSA and healthy controls. **(I–K)** Partial correlation analyses assessing the relationships between pre-sleep BOLD-CSF coupling across different regions and polysomnographic characteristics in all participants, after controlling for age, gender, education, and BMI. *p*-FDR < 0.05 indicates statistical significance. DMN, default mode network; ACC, anterior cingulate cortex; PCC, posterior cingulate cortex; AHI, apnea-hypopnea index; SpO₂, blood oxygen saturation; BOLD-CSF, blood oxygen level-dependent signal coupled to CSF signal; Glx, glutamate/glutamine; GABA, gamma-aminobutyric acid; Pre, pre-sleep; HCs, healthy controls; M-Severe OSA, moderate-to-severe OSA.

*Post hoc* comparisons showed weaker coupling in the moderate-to-severe OSA group than in HCs for both Global-BOLD-CSF coupling (Cohen’s d = 0.914 [95% CI: 0.307, 1.522], *p*-FDR *=* 0.008) and ACC-BOLD-CSF coupling (Cohen’s d = 0.837 [95% CI: 0.232, 1.442], *p*-FDR = 0.018) (Figures 3A,C). These differences remained significant after additional adjustment for PSQI score and head motion (Appendices 1, 2; Supplementary eTable 3; Supplementary eFigures 2A,B, 3A,C). The exploratory correlation analysis between global BOLD-CSF coupling and the DTI-ALPS index showed a weak association that did not reach statistical significance (*r* = 0.203, 95% CI [−0.020, 0.404], *p* = 0.060; Supplementary eFigure 4E). Additional details are provided in Appendix 3. No significant baseline differences were found for DMN-BOLD-CSF coupling or PCC-BOLD-CSF coupling (both *p*-FDR > 0.05). Following the post-sleep period, no significant inter-group differences were identified for any coupling indicator (all *p*-FDR > 0.05) (Table 2, Figures 3A–D). No significant inter-group differences were identified for GABA or Glx at either time point (all *p*-FDR > 0.05, Figures 3E–H). All MRS quality parameters are summarized in Supplementary eTable 4. Both ACC and PCC showed good voxel placement reproducibility, with mean Dice similarity coefficients > 0.80 (Appendix 4, Supplementary eTable 5, Supplementary eFigure 5) between pre-sleep and post-sleep scans. Neither the group × time interaction nor the main effect of time was significant for tissue-fraction measures in ACC or PCC, indicating that overnight changes in fCSF, fGM, and fWM did not differ across groups Supplementary eTable 6.

**Table 2:** Imaging characteristics among different groups.

Variable	HCs (*N* = 37)	Mild (*N* = 23)	M-Severe (*N* = 27)	Raw *p* value	*p*-FDR value	η_p_^2^
Glymphatic indicators						
Pre						
preGbold-csf	0.312 ± 0.145	0.288 ± 0.168^b^	0.184 ± 0.121^a^	0.009	0.038	0.110
preACC-BOLD	0.220 ± 0.104	0.204 ± 0.126	0.146 ± 0.102^a^	0.021	0.043	0.092
prePCC-BOLD	0.239 ± 0.151	0.232 ± 0.171	0.150 ± 0.108	0.104	0.104	0.055
preDMN-BOLD	0.239 ± 0.147	0.224 ± 0.174	0.150 ± 0.102	0.098	0.104	0.056
Post						
postGbold-csf	0.263 ± 0.167	0.247 ± 0.175	0.267 ± 0.150	0.640	0.640	0.012
postACC-BOLD	0.222 ± 0.143	0.184 ± 0.116	0.199 ± 0.143	0.337	0.450	0.028
postPCC-BOLD	0.239 ± 0.146	0.184 ± 0.143	0.239 ± 0.140	0.154	0.347	0.048
postDMN-BOLD	0.232 ± 0.157	0.176 ± 0.148	0.232 ± 0.139	0.174	0.347	0.045
Neurotransmitter						
Pre-ACC						
GABA/Cr	0.146 ± 0.070	0.117 ± 0.032	0.108 ± 0.034	0.189	0.549	0.042
Glx/Cr	0.216 ± 0.068	0.195 ± 0.041	0.194 ± 0.042	0.366	0.549	0.025
E/I	1.733 ± 0.756	1.810 ± 0.735	2.104 ± 1.474	0.946	0.946	0.002
Pre-PCC						
GABA/Cr	0.161 ± 0.071	0.130 ± 0.052	0.150 ± 0.080	0.133	0.399	0.051
Glx/Cr	0.168 ± 0.084	0.178 ± 0.058	0.157 ± 0.065	0.674	0.674	0.010
E/I	1.242 ± 0.745	1.656 ± 0.949	1.497 ± 1.634	0.340	0.510	0.029
Post-ACC						
GABA/Cr	0.115 ± 0.032	0.130 ± 0.032	0.111 ± 0.039	0.168	0.503	0.045
Glx/Cr	0.189 ± 0.078	0.193 ± 0.040	0.183 ± 0.053	0.839	0.839	0.005
E/I	1.886 ± 1.119	1.587 ± 0.562	1.820 ± 0.711	0.447	0.671	0.022
Post-PCC						
GABA/Cr	0.154 ± 0.073	0.173 ± 0.073	0.157 ± 0.092	0.513	0.770	0.018
Glx/Cr	0.131 ± 0.070	0.136 ± 0.062	0.132 ± 0.064	0.783	0.783	0.007
E/I	1.058 ± 0.717	1.014 ± 0.823	1.370 ± 1.435	0.379	0.770	0.027

Continuous variables are presented as mean ± SD. General linear models were performed to compare imaging and neurotransmitter measures among groups, with age, sex, education, and BMI included as covariates. HCs, healthy controls; M-Severe, moderate-to-severe obstructive sleep apnea; pre, pre-sleep; post, post-sleep; BOLD–CSF, blood oxygen level-dependent cerebrospinal fluid coupling; ACC, anterior cingulate cortex; PCC, posterior cingulate cortex; DMN, default mode network; GABA, gamma-aminobutyric acid; Glx, glutamate/glutamine; Cr, creatine; E/I, Glx/GABA; FDR, false discovery rate; η_p_^2^, partial eta squared.

a *p* < 0.05 versus the HCs group after FDR correction.

b *p* < 0.05 versus the HCs group after FDR correction.

Among all participants, AHI was negatively correlated with preGlobal-BOLD-CSF coupling (*r* = −0.382 [95% CI: −0.551, −0.184], *p*-FDR = 0.001), preDMN-BOLD-CSF coupling (*r* = −0.344 [95% CI: −0.520, −0.143], *p*-FDR = 0.002), preACC-BOLD-CSF coupling (*r* = −0.374 [95% CI: −0.539, −0.169], *p*-FDR = 0.001), and prePCC-BOLD-CSF coupling (*r* = −0.337 [95% CI: −0.514, −0.134], *p*-FDR = 0.002). Similarly, lowest SpO₂ was positively correlated with preGlobal-BOLD-CSF coupling (r = 0.369 [95% CI: 0.168, 0.541], *p*-FDR = 0.003), preDMN-BOLD-CSF coupling (*r* = 0.281 [95% CI: 0.072, 0.468], *p*-FDR = 0.015), preACC-BOLD-CSF coupling (*r* = 0.349 [95% CI: 0.139, 0.519], *p*-FDR = 0.003), and prePCC-BOLD-CSF coupling (*r* = 0.258 [95% CI: 0.047, 0.448], *p*-FDR = 0.020). Additionally, a higher arousal index was associated with lower pre-sleep BOLD-CSF coupling across all regions (*r* = −0.346 to −0.226, all *p*-FDR ≤ 0.043; Figures 3I–K). These results remained significant after adjusting for head motion (Appendix 2, Supplementary eFigures 3I–K). There were no significant correlations between pre-sleep neurochemical measures and the aforementioned PSG characteristics (Supplementary eFigure 6).

**Figure 4:**
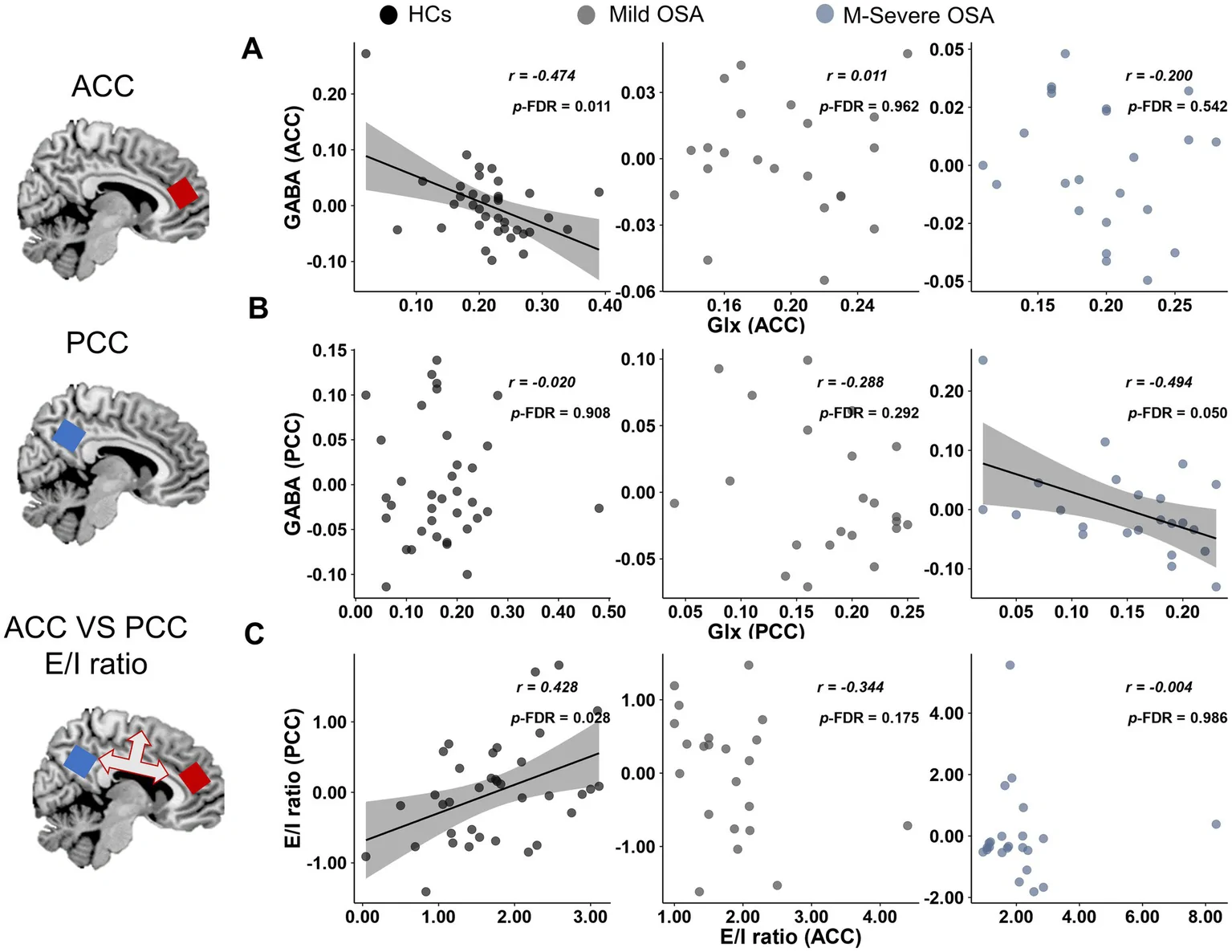
Relationships among pre-sleep Glx, GABA, and E/I ratio in the anterior and posterior cingulate cortex across groups. The Glx and GABA levels, expressed in parts per million (ppm), were measured in voxels placed in **(A)** anterior cingulate cortex (20 × 20 × 20 mm^3^), in red, and posterior cingulate cortex **(B)** (20 × 20 × 20 mm^3^) in blue. **(C)** Relationship between ACC and PCC E/I ratios across groups. *p*-FDR < 0.05 indicates statistical significance. *Glx,* Glutamate/glutamine; *GABA, G*amma-aminobutyric acid; *E/I*, *Glx*/*GABA*; AC*C,* anterior cingulate cortex; *PCC,* posterior cingulate cortex; *HCs,* healthy controls; *M-Severe OSA,* moderate-to-severe OSA.

As shown in Figure 4, partial correlation analyses revealed a significant negative correlation only in the ACC of the HCs group at pre-sleep (*r* = −0.474 [95% CI: −0.697, −0.167], *p*-FDR = 0.011), whereas no significant relationships were found in the mild OSA group (*r* = 0.011 [95% CI: −0.423, 0.441], *p*-FDR = 0.962) or the moderate-to-severe OSA group (*r* = −0.200 [95% CI: −0.573, 0.242], *p*-FDR = 0.542). For the PCC region, the moderate-to-severe OSA group exhibited a significant negative partial correlation (*r* = −0.494 [95% CI: −0.758, −0.091], *p*-FDR = 0.050), while the HCs (*r* = −0.020 [95% CI: −0.351, 0.315], *p*-FDR = 0.908) and mild OSA groups (*r* = −0.288 [95% CI: −0.640, 0.165], *p*-FDR = 0.292) did not reach significance. Regarding the E/I ratio between ACC and PCC, the HCs group showed a significant positive partial correlation (*r* = 0.428 [95% CI: 0.110, 0.666], *p*-FDR = 0.028); in contrast, the mild OSA and moderate-to-severe OSA groups showed no significant associations. These findings suggest altered regional patterns of E/I-related associations in OSA.

### 3.3 Overnight changes in BOLD-CSF coupling and excitatory-inhibitory neurochemical measures among groups

**Figure 5:**
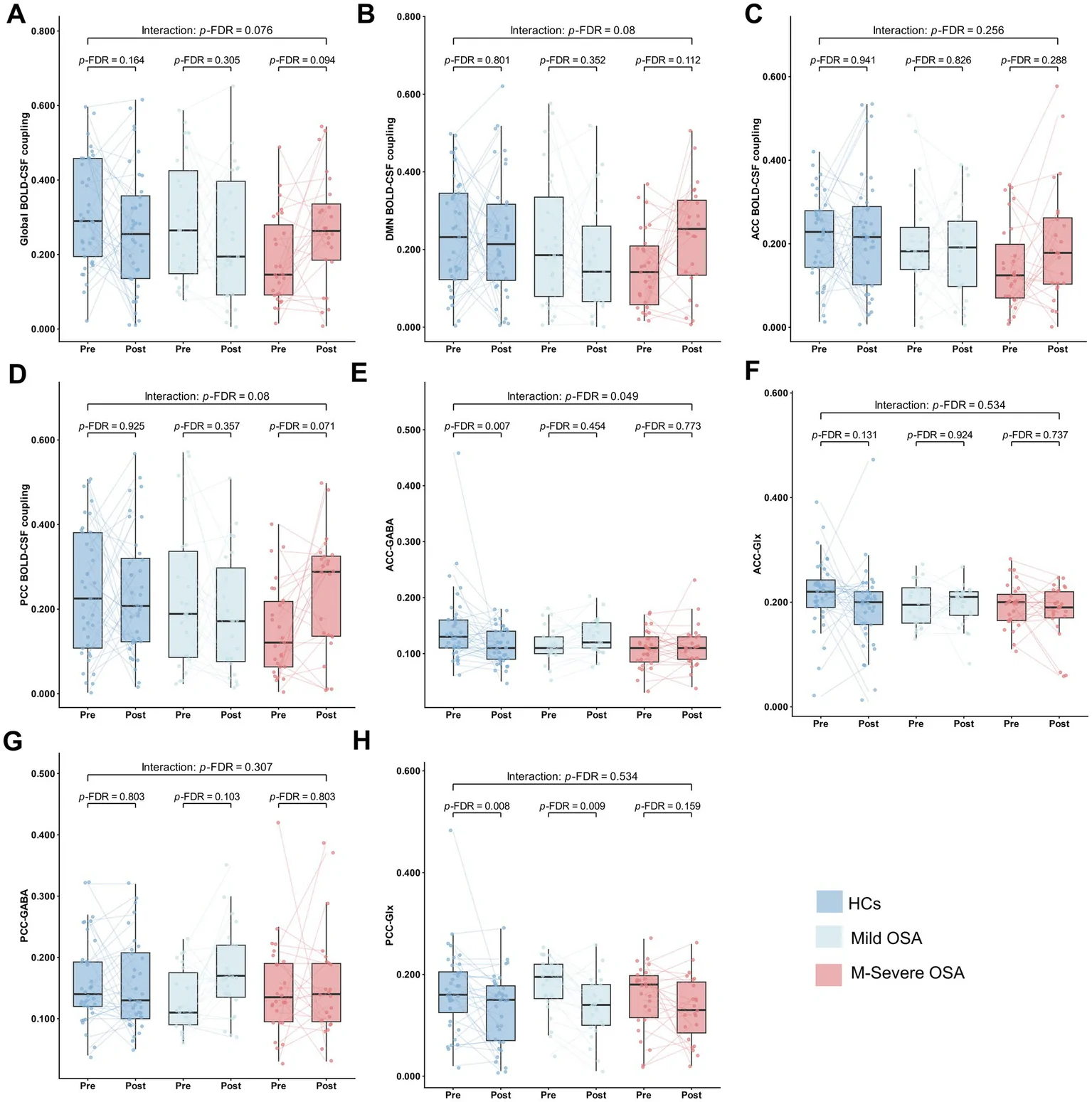
Group × time interaction effects on BOLD-CSF coupling and neurotransmitter levels. Group × time interaction effects on BOLD-CSF coupling in the global cortex, DMN, ACC, and PCC **(A–D)**. Group × time interaction effects on ACC-GABA, ACC-Glx, PCC-GABA, and PCC-Glx **(E–H)**, respectively. *P* values were adjusted within each outcome family using the Benjamini–Hochberg FDR procedure. *p*-FDR < 0.05 was considered statistically significant. *DMN,* default mode network; *ACC,* anterior cingulate cortex; *PCC,* posterior cingulate cortex; *OSA,* obstructive sleep apnea; *E/I*, Glx/GABA; *Glx,* glutamate/glutamine; *GABA,* gamma-aminobutyric acid; *HCs,* healthy controls; *M-Severe OSA,* moderate-to-severe OSA.

The linear mixed-effects models revealed nominal group × time interactions for Global-BOLD-CSF coupling (*F* = 4.161, η_p_^2^ = 0.094 [95% CI: 0.002, 0.220], raw*-p* = 0.019) and PCC-BOLD-CSF coupling (*F* = 3.202, η_p_^2^ = 0.074 [95% CI: 0.000, 0.192], raw*-p* = 0.046), together with a trend for DMN-BOLD-CSF coupling (*F* = 2.920, η_p_^2^ = 0.068 [95% CI: 0.000, 0.184], raw*-p* = 0.060). However, none of these interactions survived FDR correction (*p*-FDR = 0.076, *p*-FDR = 0.080, and *p*-FDR = 0.080, respectively). No significant group × time interaction was observed for ACC-BOLD-CSF coupling (*F* = 1.387, η_p_^2^ = 0.034 [95% CI: 0.000, 0.128], raw*-p* = 0.256, *p*-FDR = 0.256) (Figures 5A–D). All BOLD-CSF coupling group × time interactions remained nonsignificant after additional adjustment for head motion (Appendix 2, Supplementary eFigures 3E–H). A significant group × time interaction was identified for ACC-GABA after FDR correction (*F* = 4.64, η_p_^2^ = 0.105 [95% CI: 0.006, 0.235], raw-*p* = 0.012, *p*-FDR = 0.049). *Post hoc* analyses showed a significant overnight decrease in ACC-GABA among HCs (Cohen’s d = 0.75 [95% CI: 0.277–1.216], *p*-FDR = 0.007). No significant overnight changes were observed in either the mild or moderate-to-severe OSA groups (both *p*-FDR > 0.05) (Figure 5E). No significant group × time interactions were observed for PCC-GABA, ACC-Glx, or PCC-Glx (all *p*-FDR > 0.05; Figures 5F–H). Results of the ACC-GABA analyses, repeated with the arousal index, N3 sleep duration, and PSQI score added separately as covariates, are provided in Appendix 1, Supplementary eFigures 2C–E, Supplementary eTable 7. Adjustment for MRS spectral quality did not materially change the group × time effects on ACC GABA and Glx (Supplementary eTable 8).

### 3.4 Association of BOLD-CSF coupling and excitatory-inhibitory neurochemical measures with cognition in OSA patients

**Figure 6:**
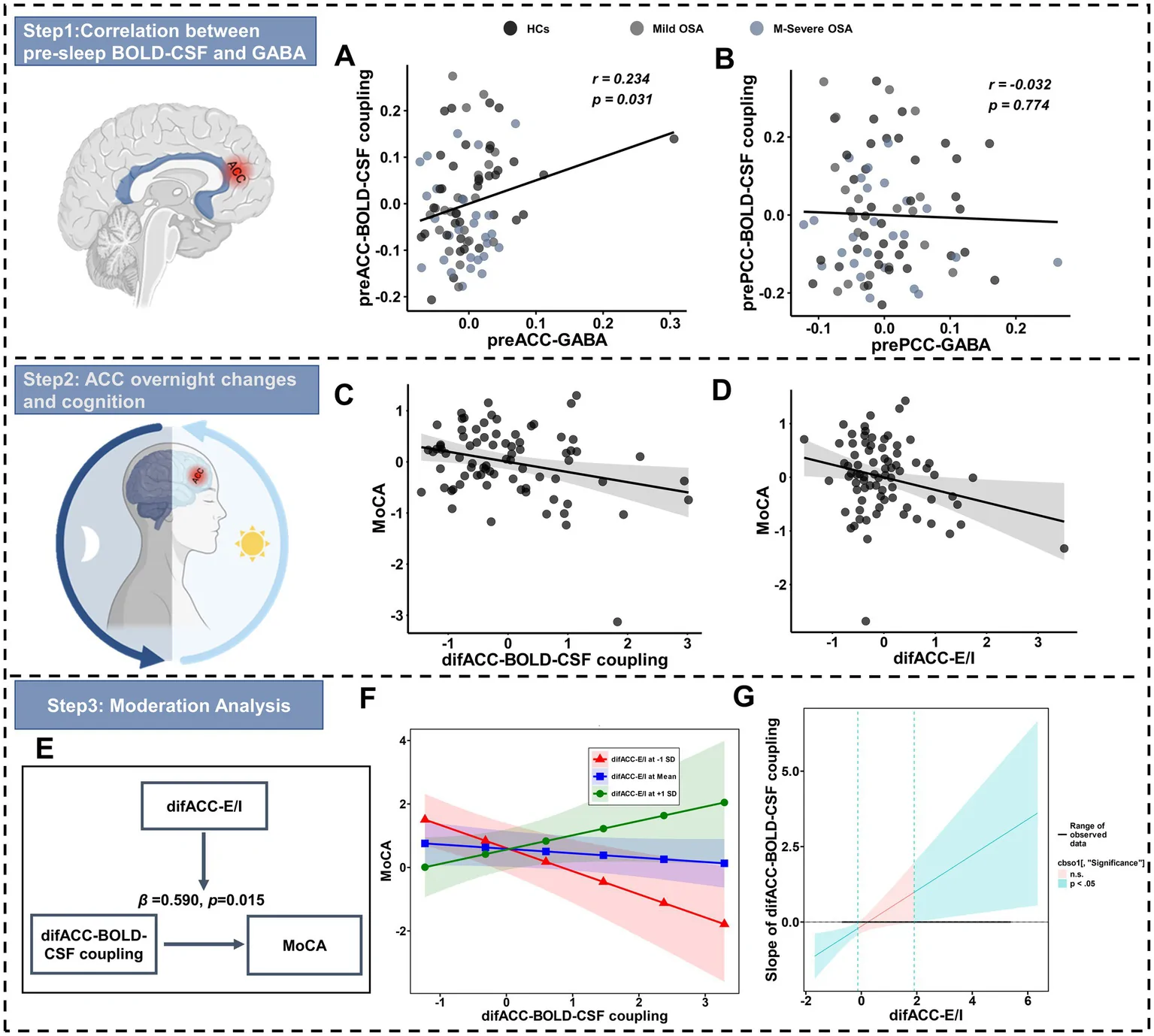
Regional specificity of the relationships among BOLD–CSF coupling, neurochemical measures, and cognitive function in patients with OSA. **(A,B)** Partial correlations between pre-sleep BOLD–CSF coupling and pre-sleep GABA levels in the ACC and PCC. **(C,D)** Associations of the magnitude of overnight variation in ACC-BOLD-CSF coupling (difACC-BOLD-CSF coupling) and ACC-E/I balance (difACC-E/I) with MoCA scores after FDR correction. **(E)** Interaction between difACC-E/I and difACC-BOLD–CSF coupling in relation to MoCA scores. **(F)** Simple slope analysis showing the association between the magnitude of overnight variation in ACC-BOLD-CSF coupling and MoCA scores at low, mean, and high magnitudes of overnight ACC-E/I change. **(G)** Johnson–Neyman analysis showing the range of overnight ACC-E/I change magnitudes over which the association between ACC-BOLD-CSF variation and MoCA scores was significant. The models were adjusted for age, sex, education, BMI, and the corresponding baseline values (preACC-BOLD–CSF coupling and preACC-E/I). *ACC*, anterior cingulate cortex; *PCC*, posterior cingulate cortex; *BOLD-CSF*, blood oxygen level-dependent–cerebrospinal fluid; *E/I*, Glx/GABA; *Glx,* glutamate/glutamine; *GABA*, gamma-aminobutyric acid; *MoCA*, Montreal Cognitive Assessment; *HCs*, healthy controls; *M-Severe OSA*, moderate-to-severe obstructive sleep apnea.

We further explored the association between BOLD-CSF coupling and neurotransmitter levels in the ACC and PCC. At pre-sleep, ACC-GABA showed a significant positive partial correlation with ACC-BOLD-CSF coupling (*r* = 0.234 [95% CI: 0.021, 0.427], *p* = 0.031). No significant correlations were observed in the PCC region (*r* = −0.032 [95% CI: −0.246, 0.185], *p* = 0.774), suggesting a regionally specific association that was evident in the ACC but not the PCC (Figures 6A,B).

In the ACC region, both difACC-BOLD-CSF coupling and difACC-E/I were significantly negatively associated with MoCA scores after FDR correction (difACC-BOLD-CSF: *β* = −0.199, 95% CI [−0.361, −0.036], *p*-FDR = 0.032; difACC-E/I: *β* = −0.235, 95% CI [−0.449, −0.021], *p*-FDR = 0.032) (Figures 6C,D). Moreover, a significant interaction was observed between difACC-BOLD-CSF coupling and difACC-E/I on MoCA scores (*β* = 0.590, 95% CI [0.121, 1.059], *p* = 0.015, ΔR^2^ = 0.062) in OSA patients (Figure 6E, Supplementary eTable 9), indicating a significant moderating effect of difACC-E/I on the association between difACC-BOLD-CSF coupling and MoCA scores. Simple slope analysis revealed that difACC-BOLD-CSF coupling was negatively associated with MoCA only at smaller magnitudes of overnight ACC E/I variation (*β* = −0.729, 95% CI [−1.181, −0.276], *p* = 0.002), but not at the mean or larger magnitudes (Figure 6F, Supplementary eTable 10). Johnson–Neyman analysis further showed that the negative association was significant when centered difACC-E/I was below −0.125, corresponding to 68.2% of the patient sample (Figure 6G). Among covariates, age was significantly associated with lower MoCA scores (*β* = −0.483, 95% CI [−0.715, −0.250], *p* < 0.001).

Individual differences in sleep quality may confound BOLD-CSF coupling and neurotransmitter measures. In addition, post-sleep ACC MRS quality differed between groups (Supplementary eTable 4). Therefore, we conducted sensitivity analyses to assess the robustness and specificity of the moderating effect. First, the moderation analysis was repeated with arousal index, N3 sleep duration, PSQI score, and post-sleep ACC MRS quality measures entered separately as additional covariates. Second, to determine whether the observed moderating effect could be attributable to either metabolite alone, we repeated the moderation analysis with difACC-Glx (dif = |post − pre|) and difACC-GABA entered separately as moderators. As shown in Supplementary eTable 11, the interaction remained significant after adjustment for sleep-related and spectral-quality covariates (*β* range, 0.554–0.710; all *p* < 0.05). In contrast, the interaction effects involving difACC-Glx (Model 2, *β* = 0.207, 95% CI [−0.068, 0.481], *p* = 0.135) and difACC-GABA (Model 3, *β* = 0.170, 95% CI [−0.161, 0.502], *p* = 0.305) were not significant. In complementary analyses using signed overnight change scores, neither the change in ACC-BOLD-CSF coupling (*β* = −0.172, 95% CI [−0.378, 0.035], *p*-FDR = 0.203) nor the change in ACC-E/I (*β* = 0.007, 95% CI [−0.236, 0.249], *p*-FDR = 0.956) was significantly associated with MoCA scores. The interaction between signed overnight changes in ACC-BOLD-CSF coupling and ACC-E/I was also not significant (*β* = −0.179, 95% CI [−0.581, 0.224], *p* = 0.373).

## 4 Discussion

The present study aimed to investigate the magnitude of overnight variation in cortical BOLD-CSF coupling and in E/I balance within the ACC and PCC of patients with OSA. We further examined their associations with cognitive performance. BOLD-CSF coupling was used as an indirect glymphatic-related imaging proxy, whereas Glx and GABA were used as neurochemical measures of E/I balance. Our findings suggest that patients with moderate-to-severe OSA exhibit reduced pre-sleep BOLD-CSF coupling, particularly in the global cortex and ACC. Moreover, the magnitudes of overnight variation in ACC-BOLD-CSF coupling and ACC-E/I balance were associated with cognitive performance. Notably, the association between BOLD-CSF coupling variation and cognition was significant only at smaller magnitudes of overnight E/I variation. This finding suggests a stronger association between cognition and BOLD-CSF coupling variation at smaller magnitudes of E/I variation.

### 4.1 Regional specificity and overnight dynamics of BOLD-CSF decoupling in OSA patients

Our findings revealed weaker pre-sleep BOLD-CSF coupling in the global cortex and ACC in patients with moderate-to-severe OSA than in HCs. In contrast, PCC and DMN BOLD-CSF coupling did not differ significantly between groups. Lower BOLD-CSF coupling was associated with higher AHI and lower values of lowest SpO₂. These findings are consistent with previously reported cortical alterations in BOLD-CSF coupling (Li Y. et al., 2025), and our results further indicate that such reduced coupling is observable in the ACC subregion. Notably, the between-group differences in coupling observed at baseline were no longer evident after sleep (Figures 3A,C). Although the group × time interactions for global, DMN, and PCC coupling did not survive FDR correction, we observed a descriptive pattern of increased post-sleep BOLD-CSF coupling in these regions among moderate-to-severe OSA patients (Figures 5A–B,D). Given the lack of statistical robustness, this pattern should be interpreted with caution and considered exploratory rather than confirmatory. One speculative interpretation is that OSA-related alterations in BOLD-CSF coupling may not reflect a fixed structural deficit, but rather a state-dependent disturbance modulated by sleep architecture, circadian phase, and arousal history. Supporting this interpretation, Wang et al. used dynamic contrast-enhanced MRI to demonstrate impaired fluid dynamics of the glymphatic drainage system in OSA, characterized by excessive perfusion with insufficient clearance (Wang et al., 2023). In parallel, previous studies have shown that sleep deprivation enhances and delays BOLD-CSF coupling. This phenomenon may reflect an interaction between compensatory upregulation and arousal-related driving mechanisms (Berberich et al., 2025; Yang et al., 2026; Zhang et al., 2026). Thus, the enhanced post-sleep coupling in OSA patients may reflect an interplay between circadian regulation and arousal responses, potentially triggering a transient boost in glymphatic-related BOLD-CSF coupling. However, BOLD-CSF coupling cannot be directly equated with quantification of glymphatic clearance and may also be influenced by arousal state, vascular pulsatility, cardiorespiratory fluctuations, and motion-related artifacts (Fultz et al., 2019; Vijayakrishnan Nair et al., 2022; Yang et al., 2022). Therefore, studies combining BOLD-CSF coupling with more direct measures of CSF flow or solute clearance in larger cohorts are needed to determine whether stronger coupling reflects enhanced glymphatic clearance (Ringstad et al., 2018; Roefs et al., 2024). In contrast, the ACC exhibited baseline decoupling and may represent a site of early decompensation, potentially attributable to the frontal cortex’s vulnerability to intermittent hypoxia. This is supported by animal and human studies showing that chronic intermittent hypoxia induces structural damage and neuronal loss in the frontal cortex of OSA models, as well as anterior brain dysfunction in OSA (Jones and Harrison, 2001; Zhang et al., 2016; Mingming et al., 2024). Taken together, our findings suggest regionally specific cortical alterations in BOLD-CSF coupling in patients with OSA. Reduced ACC-BOLD-CSF coupling was observed at pre-sleep, whereas other regions showed a descriptive trend toward increased post-sleep coupling. Further studies are needed to validate these findings.

### 4.2 Altered overnight GABA changes with ACC specificity

In this study, MRS-based findings revealed region-specific differences in the associations between excitatory and inhibitory neurochemical measures in the ACC and PCC. These association patterns varied across OSA severity groups, suggesting altered regional neurochemical relationships in OSA. We further found a significant group × time interaction for ACC-GABA levels. *Post hoc* analyses showed a decrease from evening to morning in HCs, whereas no statistically significant changes were observed in the OSA groups. These changes in HCs are consistent with recent studies showing region-specific dynamics of the GABAergic system under the combined regulation of circadian timing and sleep–wake homeostasis. These processes are important for synchronizing circadian neurons and maintaining daily rhythms in sleep–wake behavior (Hastings et al., 2023; Oishi et al., 2023; Patton et al., 2023). Meanwhile, previous multi-timepoint MEGA-PRESS studies have also demonstrated that cortical GABA levels show significant diurnal oscillations, with the ACC GABA+/Cr ratio correlating with endogenous melatonin levels in healthy young adults (Ye et al., 2024). Nevertheless, our study did not employ a multi-timepoint design. Therefore, the possible relationship between the observed GABA decline and circadian influences should be further investigated in larger studies with repeated measurements across the sleep–wake cycle.

OSA-related sleep fragmentation and intermittent hypoxia are associated with inflammation and neuroendocrine dysregulation, which may in turn alter overnight neurochemical dynamics (Šmon et al., 2023). Previous reviews have implicated E/I imbalance in OSA and its associated conditions, including sleep–wake disturbances, cognitive impairment, and mood disorders (Ralls and Cutchen, 2019; Andrisani and Andrisani, 2023; Kaczmarski et al., 2023). Our findings extend this evidence by identifying region-specific alterations in MRS-derived neurochemical measures related to E/I balance in the ACC and PCC of patients with OSA. GABA levels in patients with OSA did not significantly decrease from the pre-sleep to the post-sleep assessment. This pattern may be consistent with previous studies suggesting that elevated nocturnal GABAergic activity is associated with suppression of the ascending reticular activating system (ARAS), reduced upper airway muscle tone, and altered arousal-related respiratory regulation (Toraldo et al., 2025; Andrisani and Andrisani, 2023). However, multiple factors may contribute to the observed changes, and the observational design limits causal interpretation. Although we additionally adjusted for PSQI score, N3 sleep duration, and arousal index in exploratory sensitivity analyses, residual confounding remains possible. Notably, the group × time interaction was no longer significant after additional adjustment for the arousal index, suggesting that nocturnal sleep fragmentation may partly contribute to the observed effect. Additionally, although differences in ACC spectral quality were accounted for in sensitivity analyses, potential measurement errors due to repeated manual voxel placement cannot be entirely ruled out.

We found no significant pre-sleep differences in GABA or Glx levels between patients with OSA and controls. This finding contrasts with previous MRS studies focusing on other regions, such as the insula or thalamus. These discrepancies may stem from differences in brain regions, sample characteristics, acquisition protocols, metabolite quantification, or statistical adjustment (Gonen et al., 2020). Although our sample size was comparable to that of previous MRS studies (Sarma et al., 2016; Macey et al., 2016; Pereira et al., 2017), the relatively small sample size after severity stratification may have limited sensitivity to detect small-to-moderate effects in metabolite levels. Nevertheless, our findings suggest that repeated pre- and post-sleep measurements may provide additional information beyond single time-point comparisons. Future studies with multiple sampling time points, objective circadian markers, and more standardized MRS quality control are needed to disentangle the roles of circadian timing, sleep homeostasis, and OSA-related pathophysiology.

### 4.3 Magnitude of overnight E/I change moderates the BOLD-CSF coupling–cognition association in OSA patients

In addition, we found that GABA concentration in the ACC was positively associated with BOLD-CSF coupling at pre-sleep. This finding is consistent with previous evidence that GABA signaling may influence AQP4 expression and function, which are important for glymphatic transport (Neely et al., 2001; Li et al., 2015; Wu et al., 2023). Animal studies have further shown that GABA_A_ receptor activation can promote AQP4-dependent glymphatic transport (Wu et al., 2023). Associations between altered GABA signaling, E/I imbalance, and glymphatic dysfunction have also been reported in human studies (Tokatly Latzer et al., 2024; Pascucci et al., 2026). These findings provide a possible neurochemical context for the association between GABA levels and BOLD-CSF coupling observed in our study. However, our findings are correlational and do not establish causality. Further animal studies are needed to investigate the potential mechanisms underlying this association.

The mechanisms underlying cognitive impairment in obstructive sleep apnea (OSA) remain unclear. Glymphatic dysfunction and neurotransmitter imbalance have both been implicated in cognitive decline. In the present study, we found that greater magnitudes of overnight change in both ACC-BOLD-CSF coupling and the E/I ratio were associated with poorer cognitive performance. Moreover, the magnitude of overnight change in the E/I ratio moderated the association between BOLD-CSF coupling variation and cognition. Specifically, greater absolute overnight variation in ACC-BOLD-CSF coupling was associated with poorer cognitive performance only at smaller magnitudes of absolute ACC-E/I change. This association was not significant at the mean or larger magnitudes of E/I change. The moderation effect remained significant after additional adjustment for PSQI score, N3 sleep duration, arousal index, and post-sleep ACC spectral quality. Neither Glx nor GABA variation alone showed a significant moderating effect (Supplementary eTable 11). Although this pattern suggests that the observed interaction may relate to the combined Glx/GABA measure rather than to either metabolite considered separately, the metabolite-specific analyses should be interpreted with caution given the limited sample size. Analyses based on signed overnight changes yielded no significant associations with cognition or moderation effects, suggesting that the magnitude of overnight variation may be more relevant to the observed associations than the direction of change. Prior evidence suggests that E/I balance is a fundamental property of cortical microcircuits and varies dynamically across physiological states (Lehmann et al., 2012). Its regulation has also been linked to neuroplasticity and the acquisition of cognitive skills (Cohen Kadosh et al., 2015). Sleep is closely linked to neuroplasticity, and E/I balance may vary with sleep–wake history and circadian signals. Changes in Glu/GABA signaling may therefore contribute to cognitive maintenance during sleep disruption (Chellappa et al., 2016). In addition, Xiong et al. reported that functional network efficiency partially mediated the association between glymphatic dysfunction and cognitive impairment in patients with obstructive sleep apnea hypopnea syndrome, suggesting that alterations in functional network organization may link glymphatic dysfunction to cognitive decline (Xiong et al., 2024). Building on this evidence, we tentatively propose that the magnitude of overnight E/I variation may reflect how cortical circuits respond to the physiological stressors inherent in OSA. Within this speculative framework, larger E/I variations may reflect greater overnight neurochemical fluctuation, whereas smaller E/I changes may reflect more limited variation. Consistent with this possibility, the association between BOLD-CSF coupling variation and cognition was stronger at smaller magnitudes of E/I variation. However, this interpretation remains exploratory, as our measure captures only the magnitude of overnight change and does not directly assess circuit-level dynamics, synaptic plasticity, or compensatory mechanisms. Further studies using direct electrophysiological or pharmacological approaches are needed to clarify the relationship between E/I dynamics, BOLD-CSF coupling, and cognition in OSA. Furthermore, age emerged as a robust independent predictor of cognitive performance, consistent with established literature on age-related glymphatic degradation (Yin et al., 2025; Yoshihara et al., 2026; Yang et al., 2026). However, it should be noted that MRS-derived measures reflect the total pool of neurotransmitters within a localized brain region rather than directly measuring synaptic-level excitatory or inhibitory neurotransmission (Verstraelen et al., 2021; Bakir et al., 2026). While our results suggest that alterations in ACC-BOLD-CSF coupling and the ACC-E/I ratio were associated with cognitive performance in OSA, the precise biological mechanisms connecting these systems remain unclear. Further validation using multimodal neuroimaging and large-scale longitudinal studies is needed.

### 4.4 Limitations

This study has several limitations. First, the relatively small sample size and uneven distribution across OSA severity groups may have limited statistical power, particularly for detecting small-to-moderate effects, and may have reduced the precision and generalizability of the estimates. Larger studies with more balanced severity groups are needed to validate these preliminary findings. Second, the observational design precluded causal inference and prevented determination of the temporal relationships among BOLD-CSF coupling, E/I balance, and cognition. Longitudinal studies are needed to clarify the directionality of these associations. Third, BOLD-CSF coupling and the MRS-derived Glx/GABA ratio are indirect proxies for glymphatic dynamics and E/I balance, respectively. In the absence of molecular measures, we could not determine whether stronger BOLD-CSF coupling reflected more efficient glymphatic clearance. In addition, given the limited spectral resolution at 3 T, the present fitting procedure did not reliably separate glutamate from glutamine. MRS-based neurochemical measures also cannot distinguish intracellular from extracellular metabolite pools or directly assess synaptic-level neurotransmission. Future multimodal studies integrating BOLD-CSF coupling, diffusion-based imaging measures, tau-PET or CSF biomarkers, and higher-field MRS, such as 7 T MRS with rigorous quality control including Cramér-Rao lower-bound estimates, may help validate these measures and clarify their complementary roles. Fourth, residual confounding related to BMI imbalance, comorbidities, medication use, and other unmeasured factors cannot be excluded. Future studies should collect more comprehensive information on medical history, comorbid conditions, and medication exposure. Finally, the MoCA alone may not capture the full range of neuropsychological function. Future studies should incorporate more comprehensive neuropsychological assessments.

## 5 Conclusion

In summary, this study suggests that OSA is associated with region-specific alterations in BOLD-CSF coupling, particularly reduced ACC-BOLD-CSF coupling, together with a disrupted overnight decline in ACC-GABA. Importantly, the magnitude of overnight E/I variation moderated the association between overnight BOLD-CSF coupling variation and cognitive performance. These findings provide preliminary multimodal evidence of associations among alterations in BOLD-CSF coupling, neurochemical variation, and cognitive performance in OSA. Further studies are needed to clarify the biological mechanisms underlying these relationships.

## Data Availability

The original contributions presented in the study are included in the article/Supplementary material, further inquiries can be directed to the corresponding authors.

## Footnotes

1
http://rfmri.org/dpabi

## Glossary

ACC: Anterior cingulate cortex
AQP4: Aquaporin-4
AHI: Apnea-hypopnea index
AAL2: Automated Anatomical Labeling Atlas 2
ARAS: Ascending reticular activating system
BMI: Body mass index
BOLD-CSF coupling: Blood-oxygen-level-dependent and cerebrospinal fluid coupling
CSF: Cerebrospinal fluid
CPAP: Continuous positive airway pressure
Cr: Creatine
DMN: Default mode network
DTI-ALPS: Diffusion tensor imaging analysis along the perivascular space
E/I: Excitation/inhibition
FID: Free induction decay
FWHM: Full width at half maximum
FW-WM: Free water in white matter
FOV: Field of view
GABA: Gamma-aminobutyric acid
Glu: Glutamate
Glx: Glutamate/glutamine
MEQ: Morningness-Eveningness Questionnaire
MoCA: Montreal Cognitive Assessment
MRS: Magnetic resonance spectroscopy
MEGA-PRESS: Mescher-Garwood point-resolved spectroscopy
MQ: Memory Quotient
NAA: N-acetylaspartate
OSA: Obstructive sleep apnea
PSG: Polysomnography
PCC: Posterior cingulate cortex
PSQI: Pittsburgh Sleep Quality Index
PFC: Prefrontal cortex
PVS: Perivascular space
rs-fMRI: Resting-state functional magnetic resonance imaging
SDS: Self-Rating Depression Scale
SAS: Self-Rating Anxiety Scale
SNR: Signal-to-noise ratio
SpO₂: Peripheral oxygen saturation
UPPP: Uvulopalatopharyngoplasty
VOI: Volume of interest
WET: Water Suppression Enhanced Through T1 Effect
WMS-RC: Wechsler Memory Scale–Revised, Chinese version
