# Supplementary material for: Sleep-dependent alterations in anterior cingulate cortex excitation–inhibition balance link alterations in BOLD-CSF coupling to cognitive performance in obstructive sleep apnea
Source: Front Aging Neurosci. 2026 Sep 11;18:1932223. doi: 10.3389/fnagi.2026.1932223 (PMC13612524; doi:10.3389/fnagi.2026.1932223)
Supplement: Supplementary file 1 [file Supplementary_file_1.docx]

**Supplementary Material**

**Sleep-dependent alterations in anterior cingulate cortex excitation–inhibition balance link alterations in BOLD-CSF coupling to cognitive performance in obstructive sleep apnea**

**Appendix 1**

**Influence of sleep quality on the main findings**

Given that BOLD-CSF coupling and neurotransmitter measures may be confounded by individual differences in sleep quality, we repeated our main analyses with additional covariates. Specifically, we repeated the pre-sleep group comparisons of global and ACC-BOLD-CSF coupling, the ACC-GABA group × time interaction analysis, and the moderation analysis with additional covariates, including pre-sleep subjective sleep quality (PSQI) and objective polysomnography-derived sleep measures (arousal index and N3 sleep duration).
**1.1 Pre-sleep BOLD-CSF coupling**

As baseline BOLD-CSF coupling measures were obtained before the overnight PSG, only the PSQI score was added as an additional covariate in this analysis. Results indicated that the differences between the moderate-to-severe OSA and HC groups remained significant after additional adjustment for PSQI score, with Cohen’s d values of 0.910 (95% CI [0.297, 1.524], *p*-FDR = 0.010) for Global-BOLD-CSF coupling and 0.870 (95% CI [0.258, 1.483], *p*-FDR = 0.014) for ACC-BOLD-CSF coupling (**eFig. 2A-B, eTable 3**).

**1.2 ACC-GABA group × time interaction**

After adjusting for N3 sleep duration and PSQI score, the group × time interaction for ACC-GABA remained significant (partial η² = 0.113 and 0.114; *p*-FDR = 0.040 and 0.037, respectively). After adjustment for arousal index, the interaction was attenuated and no longer reached the FDR-corrected significance threshold, although the uncorrected association remained nominally significant (partial η² = 0.101, raw-*p* = 0.016, *p*-FDR = 0.067). Notably, across all models, HC participants consistently showed a significant overnight decrease in ACC-GABA (Cohen’s d range = 0.79−0.80; *p*‑FDR range = 0.005−0.009), whereas neither OSA subgroup showed significant changes (all *p*‑FDR > 0.4) (**eFig. 2C-E, eTable 7**). These results indicate that the overnight ACC-GABA decrease in HCs was robust to adjustment for N3 sleep duration, arousal index, and PSQI score. The attenuation of the group × time interaction after adjustment for arousal index suggests that arousal-related sleep fragmentation may partly account for the divergent overnight ACC-GABA trajectories across groups, although arousal index may also represent a component of the OSA phenotype rather than a simple external confounder (Malhotra et al., 2018).

**1.3 Moderation analysis**

In the primary model, difACC-BOLD-CSF coupling significantly interacted with difACC-E/I in relation to MoCA scores. This moderation effect remained significant after adjustment for the arousal index, N3 sleep duration, and PSQI score, with consistent positive coefficients across models (*β* range, 0.554-0.588; *p* range, 0.016-0.022; ΔR² range, 0.054-0.062) (**eTable 11**).

**Appendix 2**

**Sensitivity analyses of the influence of head motion on BOLD-CSF coupling findings**

To assess the potential confounding effect of head motion on BOLD–CSF coupling, we performed a complementary sensitivity analysis with mean framewise displacement (FD) as an additional covariate, following previous studies (Han et al., 2023). FD is a commonly used measure of head motion in neuroimaging studies (Yoo et al., 2005; Gu et al., 2020). Mean FD was computed from the six realignment parameters as the sum of the absolute temporal derivatives of the three translational and three rotational parameters (Power et al., 2012), with values averaged across each session.

After adjustment for pre-sleep mean FD, participants with moderate-to-severe OSA continued to show weaker pre-sleep Global-BOLD-CSF coupling than healthy controls (adjusted mean difference = 0.115 [95% CI: 0.023–0.208]; Cohen’s *d* = 0.797 [95% CI: 0.149–1.445]; *p*-FDR = 0.044), as well as weaker ACC-BOLD-CSF coupling (adjusted mean difference = 0.090 [95% CI: 0.022–0.158]; Cohen’s *d* = 0.843 [95% CI: 0.193–1.493]; *p*-FDR = 0.030; **eFig. 3A, C**). AHI remained negatively associated with pre-sleep BOLD–CSF coupling across all four regions: Global (*r* = −0.313 [95% CI: −0.497 to −0.101]; *p*-FDR = 0.005), DMN (*r* = −0.310 [95% CI: −0.495 to −0.098]; *p*-FDR = 0.005), ACC (*r* = −0.365 [95% CI: −0.541 to −0.160]; *p*-FDR = 0.003), and PCC (*r* = −0.312 [95% CI: −0.497 to −0.101]; *p*-FDR = 0.005; **eFig. 3I**). Lowest SpO₂ remained positively associated with BOLD–CSF coupling across all four regions (*r* = 0.226–0.333; *p*-FDR = 0.010–0.044; **eFig. 3J**). Arousal index remained negatively associated with Global-, DMN-, and PCC-BOLD–CSF coupling (**eFig. 3K**). Consistent with the primary analyses, none of the group × time interactions survived FDR correction after adjustment for time-varying mean FD (Global: *p*-FDR = 0.078; ACC: *p*-FDR = 0.248; PCC: *p*-FDR = 0.079; DMN: *p*-FDR = 0.079; **eFig. 3E–H**).

**Appendix 3**

**3.1. Exploratory cross-modal comparison between DTI-ALPS and global BOLD-CSF coupling
3.1.1 DWI data acquisition, processing, and DTI-ALPS index calculation**

The diffusion-weighted imaging (DWI) data were acquired using a spin-echo echo-planar imaging (EPI) sequence with the following parameters: repetition time (TR) = 1500 ms, echo time (TE) = 83 ms, flip angle (FA) = 90°, matrix size = 140 × 140, number of slices = 93, slice thickness = 5 mm, and voxel size = 1.5 × 1.5 × 1.5 mm³. Diffusion weighting was applied along 64 noncollinear directions with a b-value of 1000 s/mm², together with one non-diffusion-weighted volume (b = 0 s/mm²). The DWI data were further preprocessed using an integrated pipeline incorporating MRtrix3 (version 3.0.4) and FSL (version 6.0.7). Steps included denoising, Gibbs deringing, eddy current and motion correction, and B1 field inhomogeneity correction. The color-coded fractional anisotropy (FA) maps were subsequently derived using dtifit in FSL.

The DTI-ALPS index was calculated following previous studies (Taoka et al., 2017). Based on the preprocessed color-coded FA images, slices at the level of the body of the lateral ventricles were selected for ROI placement. These slices contain abundant parenchymal vessels and surrounding perivascular spaces. Spherical regions of interest (ROIs, 5 mm in diameter) were manually placed on the bilateral projection, association, and subcortical fiber areas using ITK-SNAP 3.8.0. Diffusivities along the x-axis (right-left, Dxx), y-axis (anterior-posterior, Dyy), and z-axis (inferior-superior, Dzz) were extracted and averaged across hemispheres (**eFig. 4A**). The DTI-ALPS index was then calculated as follows: $\text{}$

$$\text{DTI-ALPS }=\frac{Mean ({Dxx}_{\text{proj }}+{Dxx}_{\text{assoc }})}{Mean ({Dyy}_{\text{proj }}+{Dzz}_{\text{assoc }})}$$

To further assess the reliability of the manually derived measurements, 60 participants were randomly selected, and agreement analyses were performed between the manually calculated DTI-ALPS indices and those obtained using an open-source fully automated pipeline (Liu et al., 2024) (*https://github.com/gbarisano/alps*). The manually and automatically derived DTI-ALPS indices showed relatively good agreement, with an ICC of 0.789 (95% CI: 0.637–0.889).

**3.1.2 DTI-ALPS differences across groups and associations with PSG and gBOLD-CSF coupling**

After adjustment for age, gender, education, and BMI, the overall group effect remained significant (partial η² = 0.133, F = 6.15, *p* = 0.003) (**eFig. 4B**). Participants in the moderate-to-severe OSA group exhibited a lower mean DTI-ALPS index (1.48 ± 0.162 vs 1.68 ± 0.137; *p*-FDR = 0.003). Among all participants, the DTI-ALPS index was negatively correlated with AHI (r = −0.37 [95% CI: −0.555, −0.156]; *p*-FDR = 0.001), and positively correlated with lowest SpO₂ (r = 0.34 [95% CI: 0.119, 0.538]; *p*-FDR = 0.002) (**eFig. 4C-D**). These findings are consistent with previous DTI-ALPS studies (Lee et al., 2022; Lin et al., 2024). In the present study, gBOLD-CSF coupling analysis revealed a similar pattern in participants with moderate-to-severe OSA (**Fig. 3A, I, J**). The association between gBOLD-CSF coupling and the DTI-ALPS index was weak and did not reach statistical significance (r = 0.203 [95% CI: −0.020, 0.404], *p* = 0.060) (**eFig. 4E**). Accordingly, this result was interpreted as an exploratory cross-modal comparison rather than as evidence validating BOLD-CSF coupling as a direct measure of glymphatic function.

**3.1.3 Rationale for using BOLD-CSF coupling as a glymphatic surrogate**

Both the DTI-ALPS index and BOLD-CSF coupling are indirect imaging markers of glymphatic-related physiology rather than direct measures of glymphatic function, and neither should be regarded as a specific or validated measure of clearance. Within this framework, BOLD–CSF coupling was selected as the primary imaging measure because it was better aligned with the anatomical focus and physiological rationale of this study. Our analyses targeted cortical BOLD-CSF coupling alterations and their associations with neurochemical measurements in the anterior and posterior cingulate cortices (ACC and PCC), whereas DTI-ALPS primarily reflects water diffusivity along perivascular spaces in deep periventricular white matter. A recent study directly compared the DTI-ALPS index with intrathecal contrast-enhanced MRI, which enables more direct assessment of glymphatic tracer transport. The two measures showed limited correspondence, particularly for tracer-derived indices of cortical transport (Mossige et al., 2026). Postmortem evidence indicates that DTI-ALPS is influenced by age- and pathology-related white-matter microstructure (Li et al., 2025), limiting its specificity for cortical glymphatic transport.

The selection of BOLD-CSF coupling was also supported by the close dependence of brain fluid transport and waste clearance on sleep state. In mice, natural sleep and anesthesia expand the interstitial space, enhance cerebrospinal fluid-interstitial fluid exchange, and approximately double amyloid-β clearance compared with wakefulness (Xie et al., 2013). Human studies have further demonstrated temporally coordinated oscillations in slow-wave neural activity, cerebral haemodynamics, and cerebrospinal fluid influx during non-rapid eye movement sleep (Fultz et al., 2019). Together, these observations provide a physiological basis for using BOLD-CSF coupling as a non-invasive surrogate of sleep-related cerebrospinal fluid dynamics that is more closely aligned with our focus on cortical BOLD-CSF coupling alterations and their neurochemical correlates.

**Appendix 4**

**Reproducibility of pre- and post-sleep voxel placement**
To evaluate voxel placement reproducibility, 50 participants (50 ACC and 50 PCC voxel pairs) were randomly selected for reproducibility analysis between the two sessions. Pre- and post-sleep T1-weighted images were converted from DICOM to NIfTI format using dcm2niix (version 1.0.2). Post-sleep T1-weighted images were then rigid-body registered to the corresponding pre-sleep images using a six-degree-of-freedom transformation implemented in FSL FLIRT, without scaling or nonlinear deformation, to preserve native anatomical geometry (Jenkinson et al., 2002).

MRS voxel masks of the ACC and PCC (20 × 20 × 20 mm³) were reconstructed for all participants from the MRS DICOM geometry based on voxel position, orientation, and dimensions according to established MRS voxel localization procedures and minimum reporting recommendations. The reconstructed post-sleep volume of interest (VOI) masks were transformed into pre-sleep anatomical space using the same registration matrix. Spatial reproducibility of voxel placement was quantified using Dice similarity coefficients and centroid displacement between corresponding pre- and post-sleep VOIs. In addition, tissue composition within each VOI was calculated from T1-weighted images using SPM12 segmentation and compared between pre- and post-sleep acquisitions to evaluate potential differences in gray matter, white matter, and cerebrospinal fluid fractions.

As shown in **eTable 5** and **eFig. 5**, ACC and PCC showed comparable reproducibility (Dice: 0.834 ± 0.068 vs. 0.843 ± 0.050; displacement: 2.29 ± 1.08 mm vs. 2.16 ± 0.79 mm; both *p* > 0.05, Wilcoxon test, n = 50). After adjustment for age, gender, education, and BMI, neither the group × time interaction nor the main effect of time was significant for tissue-fraction measures in ACC or PCC after FDR correction (all *p*-FDR > 0.05; **eTable 6**). These findings indicate that overnight changes in cerebrospinal fluid fraction, gray matter fraction, and white matter fraction did not differ significantly across groups.

**eFigure 1 | Extraction of BOLD and CSF signals and quantification of BOLD-CSF couplings**

(**A**) The mean BOLD signals from cortical gray matter, DMN gray matter, ACC, and PCC were extracted to calculate BOLD-CSF coupling. The DMN was further subdivided into anterior and posterior regions corresponding to the ACC and PCC based on the AAL2 atlas (red and blue masks, respectively). The CSF ROI was placed on the bottom slice of the rs-fMRI acquisition (brightest area and red shaded area). (**B**) In a representative subject, the gBOLD signal and CSF signal showed corresponding changes of large amplitude. (**C**) The cross-correlation function between the gBOLD and CSF signals is shown in the left panel (N = 174). The gBOLD-CSF cross-correlation function showed a positive peak at a time lag of −6 s and a negative peak at a time lag of +4 s (both p < 0.001, permutation test). The right panel shows the cross-correlation function between the negative first-order derivative of the gBOLD signal and the CSF signal. (**D**) Cross-correlation functions between CSF and regional BOLD signals, as well as between CSF and the negative first-order derivatives of the regional BOLD signals, are shown for different regions. Gray shaded regions indicate the 95% confidence intervals of the mean correlation coefficients across subjects.

**eFigure 2 | Influence of sleep quality and sleep architecture on the main findings**

**(A–B)** Pre-sleep group differences in global (**A**) and anterior cingulate cortex (**B**) BOLD-CSF coupling after additional adjustment for Pittsburgh Sleep Quality Index (PSQI) score. Pairwise comparisons were corrected using the Benjamini–Hochberg false discovery rate (FDR) procedure. (**C–E**) Overnight changes in ACC GABA after additional adjustment for N3 sleep duration (**C**), PSQI score (**D**), and arousal index (**E**). All models included age, sex, education, and body mass index as covariates. *BOLD-CSF*, blood oxygen level-dependent signal coupled to CSF signal; *OSA*, obstructive sleep apnea; *HCs,* healthy controls; *M-Severe OSA,* moderate-to-severe OSA; *GABA,* gamma-aminobutyric acid; *ACC,* anterior cingulate cortex

**eFigure 3 | Sensitivity analyses of the principal findings after additional adjustment for mean framewise displacement**

**(A–D)** Adjusted group comparisons of pre-sleep BOLD-CSF coupling after additional adjustment for pre-sleep mean framewise displacement (FD). **(E–H)** Group × time interaction effects for BOLD-CSF coupling, estimated using linear mixed-effects models with time-varying mean FD included as an additional covariate. **(I–K)** Partial correlations between pre-sleep BOLD-CSF coupling across the global, ACC, PCC, and DMN regions and AHI, lowest SpO₂, and arousal index after additional adjustment for pre-sleep mean FD. All models additionally included the covariates specified in the primary analyses. *ACC*, anterior cingulate cortex; *AHI*, apnea–hypopnea index; *BOLD-CSF*, blood oxygen level-dependent signal coupled to CSF signal; *DMN*, default mode network; *HCs*, healthy controls; *OSA*, obstructive sleep apnea; *PCC*, posterior cingulate cortex; *SpO₂*, peripheral oxygen saturation; *Pre*, pre-sleep.

**eFigure 4 | DTI-ALPS index differences across groups and associations with PSG and gBOLD-CSF coupling**

**(A)** Schematic illustration of DTI-ALPS measurement. Regions of interest were placed in projection- and association-fiber areas on the fractional anisotropy color map, and directional diffusivities were used to calculate the DTI-ALPS index. **(B)** DTI-ALPS values across healthy controls (HCs) and OSA groups, adjusted for age, sex, education, and BMI. **(C-D)** Partial Spearman correlation analyses of DTI-ALPS with AHI and lowest SpO₂, controlling for age, sex, education, and BMI. *P* values were corrected using the Benjamini–Hochberg false discovery rate (FDR) method. **(E)** Spearman correlation between DTI-ALPS and gBOLD-CSF coupling. ***p*-FDR < 0.01; *NS*, not significant. *FA,* fractional anisotropy*; AHI,* apnea-hypopnea index; *SpO_2_*_,_ blood oxygen saturation; *gBOLD-CSF*, global blood-oxygen-level-dependent signal coupled to CSF signal; *DTI-ALPS,* diffusion tensor imaging analysis along the perivascular space; *HCs*, healthy controls; *M-Severe*, moderate-to-severe OSA

**eFigure 5 | Assessment of MRS voxel placement reproducibility between pre-sleep and post-sleep acquisitions**

Representative pre-sleep **(A)** and post-sleep **(B)** T1-weighted images demonstrate the placement of the ACC and PCC volumes of interest (VOIs). Post-sleep VOIs were transformed into pre-sleep anatomical space using the rigid-body registration matrix derived from T1-weighted image alignment. The overlap maps **(C)** illustrate the spatial consistency of voxel placement between the two sessions. Voxel placement reproducibility was quantified using Dice similarity coefficients. The representative participant showed excellent spatial agreement between sessions (ACC Dice = 0.942; PCC Dice = 0.946). *ACC*, anterior cingulate cortex; *PCC*, posterior cingulate cortex. Red indicates pre-sleep ACC VOI; blue indicates pre-sleep PCC VOI; green indicates post-sleep ACC VOI; yellow indicates post-sleep PCC VOI.

**eFigure 6 |** **Association between excitatory and inhibitory neurotransmitters and polysomnographic features in the ACC and PCC**

No significant correlations were found between pre-sleep neurochemical measures and PSG characteristics. *p-*FDR < 0.05 indicates statistical significance. *AHI*, apnea-hypopnea index; *HCs,* healthy controls; *M-Severe OSA,* moderate-to-severe OSA; *SpO2*, blood oxygen saturation; *GABA*, gamma-aminobutyric acid; *Glx*, glutamate/glutamine; *ACC*, anterior cingulate cortex; *PCC*, posterior cingulate cortex; *Pre,* pre-sleep.

**eTable 1** **The components for each cognitive domain of MoCA.**

| **Cognitive domain** | **Total score** | **MoCA items** |
| --- | --- | --- |
| Memory | 12 | Delayed recall (5) |
|  |  | Orientation (6) |
|  |  | Forward digit span (1) |
| Visuospatial | 4 | Cube copy (1) |
|  |  | Clock drawing (3) |
| Language | 5 | Animal picture naming (3) |
|  |  | Sentence repetition (2) |
| Attention | 4 | Serial‑7 subtraction (3) |
|  |  | Vigilance / Tapping test (1) |
| Executive | 5 | Backward digit span (1) |
|  |  | Trail Making Test (1) |
|  |  | Abstraction (2) |
|  |  | Letter fluency (1) |

*Note:* *MoCA,* Montreal Cognitive Assessment

**eTable2 Magnetic Resonance Spectroscopy reporting checklist**

| 1. Hardware |  |
| --- | --- |
| a. Field strength [T] | 3.0T |
| b. Manufacturer | United Imaging Healthcare, Shanghai, China |
| c. Model (software version if available) | uMR790, United Imaging Healthcare (UIH), software version V10_3T |
| d. RF coils: nuclei (transmit/receive), number of channels, type, body part | ¹H, 32-channel, head coil (United Imaging Healthcare) |
| e. Additional hardware | Foam cushions or vacuum headrests to minimize motion |
| 2. Acquisition |  |
| a. Pulse sequence | MEGA-PRESS (MEshcher-GArwood Point RESolved Spectroscopy) |
| b. Volume of Interest (VOI) locations | Anterior and posterior cingulate cortices (ACC and PCC) |
| c. Nominal VOI size [cm³, mm³] | VOI size =20*20*20 mm^3^ |
| d. Repetition Time (TR), Echo Time (TE) [ms, s] | TR = 2000 ms, TE = 69 ms |
| e. Total number of excitations or acquisitions per spectrum in time series for kinetic studies  i. Number of averaged spectra per time-point ii. Averaging method (e.g. block-wise or moving average) iii. Total number of spectra (acquired / in time-series) | i. Number of averaged spectra per time-point: 100 “ON” + 100 “OFF” averages  ii. Averaging method: Block-wise averaging (MEGA-PRESS default)  iii. Total number of spectra: 2 (pre- and post-sleep) per region; total 4 per subject |
| f. Additional sequence parameters (spectral width in Hz, number of spectral points, frequency offsets)  If STEAM:, Mixing Time (TM)  If MRSI: 2D or 3D, FOV in all directions, matrix size, acceleration factors, sampling method | Spectral width: 2000 Hz  Data points: 1024  Frequency offsets: 1.9 ppm (editing ON), 7.5 ppm (editing OFF)  STEAM / MRSI: Not applicable (MEGA-PRESS single voxel acquisition used) |
| g. Water Suppression Method | Water Suppression Enhanced Through T1 Effect (WET) |
| h. Shimming Method, reference peak, and thresholds for “acceptance of shim” chosen | Shimming method: Automated Dynamic Frequency Calibration  Reference peak: Cr  Acceptance threshold: FWHM < 7 Hz |
| i. Triggering or motion correction method (respiratory, peripheral, cardiac triggering, incl. device used and delays) | None applied; head motion minimized with foam padding. |
| 3. Data analysis methods and outputs |  |
| a. Analysis software | Vendor-supplied Spectroscopy Analysis Tool in uWS-MR workstation (UIH, version 2.1). |
| b. Processing steps deviating from quoted reference or product | None. All spectra were processed using the vendor-supplied uWS-MR workstation analysis module with default parameters. |
| c. Output measure (e.g. absolute concentration, institutional units, ratio) | Spectral peak integral area and metabolite ratio |
| d. Quantification references and assumptions, fitting model assumptions | Vendor-supplied basis fitting model with Gaussian line-shape assumptions. No external tissue correction applied |
| 4. Data Quality |  |
| a. Reported variables (SNR, Linewidth (with reference peaks)) | FWHM (NAA) < 7 Hz  SNR and line-fitting reviewed visually  Reference peaks: Water (scaling), NAA (linewidth) |
| b. Data exclusion criteria | FWHM > 7 Hz (NAA peak)  Spectra with artifacts or poor fit excluded after visual inspection |
| c. Quality measures of postprocessing: Model fitting (e.g., CRLB, goodness of fit, SD of residual) | FWHM (NAA) < 7 Hz  SNR (NAA) < 10  Visual inspection of fit and residual  No SD of residual reported numerically |
| d. Sample Spectrum | Figure 2 |

**eTable 3** **Sensitivity analyses of pre-sleep global and ACC-BOLD-CSF coupling after adjustment for PSQI**

| **Indicator** | **Model** | **Cohen’s d** | **95% CI** | ***p*-FDR** |
| --- | --- | --- | --- | --- |
| Global-BOLD-CSF coupling | Original model | 0.914 | [0.307, 1.522] | 0.008 |
|  | Original + PSQI | 0.910 | [0.297, 1.524] | 0.010 |
| ACC-BOLD-CSF coupling | Original model | 0.837 | [0.232, 1.442] | 0.018 |
|  | Original + PSQI | 0.870 | [0.258, 1.483] | 0.014 |

*Note:* The original models were adjusted for age, sex, education, and BMI. Sensitivity models were additionally adjusted for the Pittsburgh Sleep Quality Index (PSQI). Cohen’s d represents the standardized effect size. *ACC*, anterior cingulate cortex; *BOLD-CSF*, blood oxygen level-dependent signal coupled to CSF signal.

**eTable 4 MRS quality parameters after covariate adjustment**

| **Metric** | **HCs (n = 37)** | **Mild OSA (n = 23)** | **M-Severe OSA (n = 27)** | **F** | ***p*-FDR** |
| --- | --- | --- | --- | --- | --- |
| **preACC** |  |  |  |  |  |
| NAA SNR | 103.02 ± 11.93 | 147.85 ± 14.06 | 132.42 ± 14.75 | 2.893 | 0.168 |
| NAA FWHM (Hz) | 5.49 ± 0.16 | 5.53 ± 0.19 | 6.08 ± 0.20 | 2.548 | 0.168 |
| Cr SNR | 87.38 ± 10.73 | 132.71 ± 12.64 | 117.77 ± 13.26 | 3.670 | 0.168 |
| Cr FWHM (Hz) | 6.68 ± 0.27 | 6.16 ± 0.31 | 7.01 ± 0.33 | 1.939 | 0.203 |
| GABA SNR | 5.25 ± 0.59 | 6.76 ± 0.70 | 6.92 ± 0.73 | 1.739 | 0.203 |
| GABA FWHM (Hz) | 2.95 ± 0.20 | 3.15 ± 0.24 | 3.24 ± 0.25 | 0.352 | 0.705 |
| Glx SNR | 14.79 ± 1.54 | 19.36 ± 1.81 | 17.58 ± 1.90 | 1.795 | 0.203 |
| Glx FWHM (Hz) | 5.37 ± 0.22 | 4.78 ± 0.26 | 5.28 ± 0.27 | 1.730 | 0.203 |
| **prePCC** |  |  |  |  |  |
| NAA SNR | 92.97 ± 12.30 | 138.22 ± 14.50 | 131.22 ± 15.20 | 2.982 | 0.232 |
| NAA FWHM (Hz) | 5.57 ± 0.10 | 5.44 ± 0.12 | 5.67 ± 0.12 | 1.059 | 0.353 |
| Cr SNR | 80.93 ± 11.25 | 123.51 ± 13.26 | 111.00 ± 13.90 | 2.972 | 0.232 |
| Cr FWHM (Hz) | 6.36 ± 0.10 | 6.21 ± 0.12 | 6.45 ± 0.13 | 1.024 | 0.365 |
| GABA SNR | 5.09 ± 0.64 | 6.17 ± 0.75 | 5.80 ± 0.79 | 0.592 | 0.592 |
| GABA FWHM (Hz) | 3.24 ± 0.21 | 3.24 ± 0.24 | 3.41 ± 0.26 | 0.143 | 0.867 |
| Glx SNR | 11.90 ± 1.75 | 18.09 ± 2.06 | 14.47 ± 2.16 | 2.607 | 0.324 |
| Glx FWHM (Hz) | 5.00 ± 0.23 | 4.98 ± 0.27 | 4.89 ± 0.28 | 0.039 | 0.962 |
| **postACC** |  |  |  |  |  |
| NAA SNR | 91.98 ± 11.54 | 130.95 ± 13.60 | 104.32 ± 14.26 | 2.458 | 0.182 |
| NAA FWHM (Hz) | 5.47 ± 0.13 | 5.64 ± 0.15 | 5.80 ± 0.16 | 1.058 | 0.353 |
| Cr SNR | 79.66 ± 10.07 | 116.00 ± 11.87 | 95.18 ± 12.45 | 2.690 | 0.182 |
| Cr FWHM (Hz) | 6.41 ± 0.18 | 6.47 ± 0.21 | 6.97 ± 0.22 | 1.940 | 0.203 |
| GABA SNR | 5.10 ± 0.53 | 6.86 ± 0.63 | 5.98 ± 0.66 | 2.246 | 0.182 |
| GABA FWHM (Hz) | 2.57 ± 0.19 | 3.39 ± 0.22 | 2.95 ± 0.23 | 3.950 | 0.096 |
| Glx SNR | 13.54 ± 1.59 | 22.06 ± 1.88 | 15.49 ± 1.97 | 6.468 | 0.024 |
| Glx FWHM (Hz) | 4.72 ± 0.24 | 5.21 ± 0.28 | 5.37 ± 0.29 | 1.438 | 0.245 |
| **postPCC** |  |  |  |  |  |
| NAA SNR | 82.03 ± 10.87 | 133.35 ± 12.81 | 109.31 ± 13.43 | 4.532 | 0.037 |
| NAA FWHM (Hz) | 5.44 ± 0.09 | 5.56 ± 0.11 | 5.80 ± 0.11 | 2.652 | 0.104 |
| Cr SNR | 69.26 ± 9.98 | 109.76 ± 11.77 | 95.42 ± 12.34 | 3.365 | 0.055 |
| Cr FWHM (Hz) | 6.33 ± 0.16 | 6.24 ± 0.19 | 6.74 ± 0.19 | 1.904 | 0.157 |
| GABA SNR | 4.77 ± 0.98 | 8.36 ± 1.15 | 4.97 ± 1.21 | 3.448 | 0.076 |
| GABA FWHM (Hz) | 7.97 ± 13.98 | 23.70 ± 16.48 | 25.87 ± 17.28 | 0.347 | 0.708 |
| Glx SNR | 9.48 ± 1.80 | 20.72 ± 2.12 | 14.24 ± 2.23 | 8.060 | 0.008 |
| Glx FWHM (Hz) | 4.14 ± 0.23 | 5.06 ± 0.27 | 5.20 ± 0.28 | 4.605 | 0.037 |

*Note:* Data are presented as adjusted estimated marginal means ± standard errors. Group differences were assessed using general linear models adjusted for age, sex, education, and BMI, followed by false discovery rate (FDR) correction for multiple comparisons. *ACC*, anterior cingulate cortex; *PCC*, posterior cingulate cortex; *HCs*, healthy controls; *M-Severe*, moderate-to-severe obstructive sleep apnea; *NAA*, N-acetylaspartate; *Cr*, creatine; *GABA*, gamma-aminobutyric acid; *Glx*, glutamate/glutamine; *SNR*, signal-to-noise ratio; *FWHM*, full width at half maximum of the fitted spectral peak; *Hz*, hertz.

**eTable 5 Voxel placement reproducibility between pre-sleep and post-sleep MRS acquisitions in the ACC and PCC**

| **Region** | **Dice coefficient** | **Centroid displacement (mm)** | **Dice ≥ 0.8, n (%)** |
| --- | --- | --- | --- |
| ACC | 0.834 ± 0.068 | 2.29 ± 1.08 | 35 (70.0%) |
| PCC | 0.843 ± 0.050 | 2.16 ± 0.79 | 41 (82.0%) |
| *p* value | 0.483 | 0.567 | — |

*Note:* Data are presented as mean ± standard deviation (SD). Dice similarity coefficients were calculated to assess spatial overlap between corresponding pre-sleep and post-sleep volumes of interest (VOIs). Centroid displacement represents the Euclidean distance between the geometric centers of corresponding VOIs after registration. *ACC*, anterior cingulate cortex; *PCC*, posterior cingulate cortex

**eTable 6 Overnight changes in tissue composition within MRS volumes of interest**

| **ROI** | **Measure** (%) | **Effect** | **F** | ***p*-FDR** | **η_p_²** | **95% CI** |
| --- | --- | --- | --- | --- | --- | --- |
| ACC | fCSF | group × time | 0.010 | 0.991 | <0.001 | [0, <0.001] |
| ACC | fGM | group × time | 1.101 | 0.991 | 0.028 | [0, 0.119] |
| ACC | fWM | group × time | 0.362 | 0.991 | 0.009 | [0, 0.072] |
| PCC | fCSF | group × time | 1.877 | 0.481 | 0.048 | [0, 0.158] |
| PCC | fGM | group × time | 0.958 | 0.583 | 0.025 | [0, 0.115] |
| PCC | fWM | group × time | 0.236 | 0.790 | 0.006 | [0, 0.061] |
| ACC | fCSF | time | 0.304 | 0.875 | 0.004 | [0, 0.075] |
| ACC | fGM | time | 0.968 | 0.875 | 0.012 | [0, 0.101] |
| ACC | fWM | time | 0.001 | 0.979 | <0.001 | [0, <0.001] |
| PCC | fCSF | time | 4.247 | 0.129 | 0.054 | [0, 0.181] |
| PCC | fGM | time | 1.336 | 0.377 | 0.018 | [0, 0.116] |
| PCC | fWM | time | 0.043 | 0.836 | <0.001 | [0, 0.049] |

*Note:* Tissue fractions within MRS volumes of interest (VOIs) were calculated from T1-weighted image segmentation and compared between pre-sleep and post-sleep acquisitions using linear mixed-effects models adjusted for age, sex, education, and BMI. *p* values were corrected using the Benjamini–Hochberg false discovery rate (FDR) procedure. *fCSF*, cerebrospinal fluid fraction; *fGM*, gray matter fraction; *fWM*, white matter fraction; *VOI*, volume of interest; *ACC*, anterior cingulate cortex; *PCC*, posterior cingulate cortex; *ηp²*, partial eta squared.

**eTable 7 Sensitivity analyses of the ACC GABA group × time interaction after adjustment for sleep-related factors**

| **Model** | **Additional covariate** | **F** | **Raw *p*** | ***p*-FDR** | **η_p_²** | **95% CI** |
| --- | --- | --- | --- | --- | --- | --- |
| Model 1 | + Arousal index | 4.32 | 0.016 | 0.067 | 0.101 | [0.003, 0.231] |
| Model 2 | + N3 sleep duration | 4.88 | 0.010 | 0.040 | 0.113 | [0.008, 0.246] |
| Model 3 | + PSQI score | 4.97 | 0.009 | 0.037 | 0.114 | [0.008, 0.248] |

*Note:* All models included age, sex, education, and BMI as covariates. Models 1–3 were additionally adjusted for the corresponding sleep-related covariate listed in the table. *p* values were corrected using the Benjamini–Hochberg false discovery rate (FDR) procedure. *ACC*, anterior cingulate cortex; *GABA*, gamma-aminobutyric acid; *PSQI*, Pittsburgh Sleep Quality Index; *N3*, stage N3 sleep; *ηp²*, partial eta squared.

**eTable 8 Sensitivity analyses of ACC GABA and Glx group × time effects after adjustment for spectral quality**

| **Model** | **Covariates included** | **Effect tested** | **F** | ***p* value** | **η_p_²** | **95% CI** |
| --- | --- | --- | --- | --- | --- | --- |
| **ACC GABA** |  |  |  |  |  |  |
| Model 1 | Age, sex, education, BMI | group × time | 4.64 | 0.0124 | 0.105 | [0.006, 0.235] |
| Model 2 | Model 1 + SNR + FWHM | group × time | 4.54 | 0.0137 | 0.111 | [0.005, 0.240] |
| Model 3 | Model 2 + SNR × time + FWHM × time | group × time | 4.31 | 0.0171 | 0.108 | [0.003, 0.239] |
| **ACC Glx** |  | group × time |  |  |  |  |
| Model 4 | Age, sex, education, BMI | group × time | 0.78 | 0.463 | 0.019 | [0.000, 0.096] |
| Model 5 | Model 4 + SNR + FWHM | group × time | 0.09 | 0.912 | 0.002 | [0.000, 0.032] |
| Model 6 | Model 5 + SNR × time + FWHM × time | group × time | 0.18 | 0.839 | 0.005 | [0.000, 0.050] |

*Note:* Models 1 and 4 were the original interaction models for ACC GABA and ACC Glx, respectively, and included age, sex, education, and BMI as covariates. Models 2 and 5 additionally included SNR and FWHM. Models 3 and 6 further included the SNR × time and FWHM × time interaction terms. *CI*, confidence interval; *SNR*, signal-to-noise ratio; *FWHM*, full width at half maximum; *ηp²*, partial eta-squared.

**eTable 9 Moderating effect of difACC-E/I on the association between difACC-BOLD-CSF coupling and MoCA scores**

| **Term** | ***β*** | **SE** | **95% CI** | **t value** | ***p*-value** |
| --- | --- | --- | --- | --- | --- |
| difACC-BOLD-CSF coupling × difACC-E/I | 0.590 | 0.231 | [0.121, 1.059] | 2.557 | **0.015** |
| difACC-BOLD-CSF coupling (X) | −0.139 | 0.110 | [−0.362, 0.085] | −1.261 | 0.216 |
| difACC-E/I (W) | −0.025 | 0.224 | [−0.480, 0.431] | −0.109 | 0.914 |
| age | −0.483 | 0.115 | [−0.715, −0.250] | −4.211 | **<0.001** |
| gender | −0.616 | 0.335 | [−1.297, 0.065] | −1.839 | 0.075 |
| education | 0.227 | 0.123 | [−0.023, 0.478] | 1.843 | 0.074 |
| BMI | 0.162 | 0.110 | [−0.062, 0.385] | 1.469 | 0.151 |
| pre-sleep ACC-BOLD-CSF coupling | 0.008 | 0.109 | [−0.214, 0.230] | 0.076 | 0.940 |
| pre-sleep ACC-E/I | 0.043 | 0.212 | [−0.389, 0.474] | 0.201 | 0.842 |

*Note:* dif variables were calculated as absolute-change scores (dif = |post − pre|), reflecting the magnitude of overnight change. The significant difACC-BOLD-CSF coupling × difACC-E/I interaction indicates a moderating effect of difACC-E/I on the association between difACC-BOLD-CSF coupling and MoCA scores. *β* represents standardized regression coefficients. Bold *p*-values indicate statistical significance (*p* < 0.05). *ACC*, anterior cingulate cortex; *BOLD-CSF,* blood oxygen level-dependent signal coupled to cerebrospinal fluid signal; *E/I*, Glx/GABA; *MoCA*, Montreal Cognitive Assessment; *BMI*, body mass index.

**eTable 10 Simple slopes of difACC-BOLD-CSF coupling on MoCA at different magnitudes of ACC E/I change**

| **difACC-E/I** | **Simple Slope (β)** | **SE** | **95% CI** | **t value** | ***p*-value** |
| --- | --- | --- | --- | --- | --- |
| -1 SD (smaller magnitude) | −0.729 | 0.223 | [−1.181, −0.276] | −3.273 | **0.002** |
| Mean (0) | −0.139 | 0.110 | [−0.362, 0.085] | −1.261 | 0.216 |
| +1 SD (larger magnitude) | 0.452 | 0.285 | [−0.127, 1.031] | 1.586 | 0.122 |

*Note:* dif variables were calculated as absolute-change scores (dif = |post − pre|), reflecting the magnitude of overnight change. Simple slope analyses examined the association between difACC-BOLD-CSF coupling and MoCA at smaller (−1 SD), mean, and larger (+1 SD) magnitudes of difACC-E/I. A significant negative association was observed only at the smaller magnitude of difACC-E/I. Bold *p*-values indicate statistical significance (p < 0.05). *β* represents standardized regression coefficients. *ACC*, anterior cingulate cortex; *E/I*, Glx/GABA; *MoCA*, Montreal Cognitive Assessment; *SD*, standard deviation.

**eTable 11 Sensitivity analyses of the difACC-BOLD-CSF coupling × difACC-E/I interaction**

| **Model** | ***β*** | **SE** | **95% CI** | **t value** | ***p* value** |
| --- | --- | --- | --- | --- | --- |
| Primary model (difACC-E/I) | 0.590 | 0.231 | [0.121, 1.059] | 2.557 | 0.015 |
| + Arousal index | 0.588 | 0.233 | [0.114, 1.062] | 2.526 | 0.017 |
| + N3 sleep duration | 0.554 | 0.231 | [0.084, 1.024] | 2.396 | 0.022 |
| + PSQI | 0.580 | 0.228 | [0.115, 1.045] | 2.540 | 0.016 |
| + Post-sleep ACC Glx SNR | 0.590 | 0.234 | [0.113, 1.066] | 2.518 | 0.017 |
| + Post-sleep ACC Glx FWHM | 0.567 | 0.230 | [0.100, 1.035] | 2.468 | 0.019 |
| + Post-sleep ACC GABA SNR | 0.710 | 0.228 | [0.245, 1.175] | 3.116 | 0.004 |
| + Post-sleep ACC GABA FWHM | 0.616 | 0.224 | [0.119, 1.114] | 2.526 | 0.017 |
| Model 2 (difACC-Glx) | 0.207 | 0.135 | [−0.068, 0.481] | 1.528 | 0.135 |
| Model 3 (difACC-GABA) | 0.170 | 0.164 | [−0.161, 0.502] | 1.042 | 0.305 |

*Note*: dif variables were calculated as absolute-change scores (dif = |post − pre|), reflecting the magnitude of overnight change. The primary model included age, gender, education, BMI, pre-sleep ACC-BOLD-CSF coupling, and pre-sleep ACC E/I ratio as covariates. Sensitivity models additionally included each sleep-related or MRS quality covariate separately, as specified in the table. Models 2 and 3 used the corresponding pre-sleep ACC Glx and pre-sleep ACC GABA measures as baseline covariates, while retaining the other covariates used in the primary model. *β* represents the standardized regression coefficient. *ACC*, anterior cingulate cortex; *BOLD-CSF*, blood oxygen level-dependent signal coupled to cerebrospinal fluid signal; *E/I*, Glx/GABA; *GABA*, gamma-aminobutyric acid; *Glx*, glutamate/glutamine; *FWHM*, full width at half maximum; *SNR*, signal-to-noise ratio; *PSQI*, Pittsburgh Sleep Quality Index.

**References**

Fultz, N. E., Bonmassar, G., Setsompop, K., Stickgold, R. A., Rosen, B. R., Polimeni, J. R., et al. (2019). Coupled electrophysiological, hemodynamic, and cerebrospinal fluid oscillations in human sleep. *Science* 366, 628–631. doi: 10.1126/science.aax5440

Gu, Y., Han, F., Sainburg, L. E., and Liu, X. (2020). Transient Arousal Modulations Contribute to Resting-State Functional Connectivity Changes Associated with Head Motion Parameters. *Cereb. Cortex* 30, 5242–5256. doi: 10.1093/cercor/bhaa096

Han, F., Liu, X., Mailman, R. B., Huang, X., and Liu, X. (2023). Resting-state global brain activity affects early β-amyloid accumulation in default mode network. *Nat. Commun.* 14, 7788. doi: 10.1038/s41467-023-43627-y

Jenkinson, M., Bannister, P., Brady, M., and Smith, S. (2002). Improved optimization for the robust and accurate linear registration and motion correction of brain images. *NeuroImage* 17, 825–841. doi: 10.1016/s1053-8119(02)91132-8

Lee, H.-J., Lee, D. A., Shin, K. J., and Park, K. M. (2022). Glymphatic system dysfunction in obstructive sleep apnea evidenced by DTI-ALPS. *Sleep Med.* 89, 176–181. doi: 10.1016/j.sleep.2021.12.013

Li, S., Chen, R., Cao, Z., Zhu, Q., Ma, Y., Zhu, K., et al. (2025). Microstructural Bias in the Assessment of Periventricular Flow as Revealed in Postmortem Brains. *Radiology* 316, e250753. doi: 10.1148/radiol.250753

Lin, S., Lin, X., Chen, S., Liang, Q., Li, Y., Wei, F., et al. (2024). Association of MRI Indexes of the Perivascular Space Network and Cognitive Impairment in Patients with Obstructive Sleep Apnea. *Radiology* 311, e232274. doi: 10.1148/radiol.232274

Liu, X., Barisano, G., Shao, X., Jann, K., Ringman, J. M., Lu, H., et al. (2024). Cross-Vendor Test-Retest Validation of Diffusion Tensor Image Analysis along the Perivascular Space (DTI-ALPS) for Evaluating Glymphatic System Function. *Aging Dis.* 15, 1885–1898. doi: 10.14336/AD.2023.0321-2

Malhotra, R. K., Kirsch, D. B., Kristo, D. A., Olson, E. J., Aurora, R. N., Carden, K. A., et al. (2018). Polysomnography for Obstructive Sleep Apnea Should Include Arousal-Based Scoring: An American Academy of Sleep Medicine Position Statement. *J. Clin. Sleep Med. JCSM Off. Publ. Am. Acad. Sleep Med.* 14, 1245–1247. doi: 10.5664/jcsm.7234

Mossige, I., Valnes, L. M., Storås, T. H., Emblem, K. E., Eide, P. K., and Ringstad, G. (2026). Comparing Glymphatic Function Measures: Diffusion Tensor Image Analysis Along Perivascular Spaces (DTI-ALPS) versus Intrathecal Contrast-Enhanced MRI. *Radiology* 318, e252070. doi: 10.1148/radiol.252070

Power, J. D., Barnes, K. A., Snyder, A. Z., Schlaggar, B. L., and Petersen, S. E. (2012). Spurious but systematic correlations in functional connectivity MRI networks arise from subject motion. *NeuroImage* 59, 2142–2154. doi: 10.1016/j.neuroimage.2011.10.018

Taoka, T., Masutani, Y., Kawai, H., Nakane, T., Matsuoka, K., Yasuno, F., et al. (2017). Evaluation of glymphatic system activity with the diffusion MR technique: diffusion tensor image analysis along the perivascular space (DTI-ALPS) in Alzheimer’s disease cases. *Jpn. J. Radiol.* 35, 172–178. doi: 10.1007/s11604-017-0617-z

Xie, L., Kang, H., Xu, Q., Chen, M. J., Liao, Y., Thiyagarajan, M., et al. (2013). Sleep Drives Metabolite Clearance from the Adult Brain. Science 342, 373–377. doi: 10.1126/science.1241224

Yoo, S.-S., Choi, B.-G., Juh, R., Pae, C.-U., and Lee, C.-U. (2005). Head motion analysis during cognitive fMRI examination: application in patients with schizophrenia. *Neurosci. Res.* 53, 84–90. doi: 10.1016/j.neures.2005.06.004
